# Hydrophobic Microenvironment Modulation of Ru Nanoparticles in Metal–Organic Frameworks for Enhanced Electrocatalytic N_2_ Reduction

**DOI:** 10.1002/advs.202405210

**Published:** 2024-07-10

**Authors:** Lulu Wen, Xiaoshuo Liu, Xinyang Li, Hanlin Zhang, Shichuan Zhong, Pan Zeng, Syed Shoaib Ahmad Shah, Xiaoye Hu, Weiping Cai, Yue Li

**Affiliations:** ^1^ Key Lab of Materials Physics Anhui Key Lab of Nanomaterials and Nanotechnology Institute of Solid State Physics Hefei Institutes of Physical Science Chinese Academy of Sciences Hefei Anhui 230031 P. R. China; ^2^ Key Laboratory of Energy Thermal Conversion and Control of Ministry of Education School of Energy and Environment Southeast University Nanjing 210096 P. R. China; ^3^ University of Science and Technology of China Hefei 230026 P. R. China; ^4^ Department of Chemistry School of Natural Sciences National University of Sciences and Technology Islamabad 44000 Pakistan; ^5^ School of Physical Science and Technology Tiangong University Tianjin 300387 P. R. China

**Keywords:** *d*‐band center, electrocatalytic N_2_ reduction, hydrophobic microenvironment, metal–organic frameworks, Ru nanoparticles

## Abstract

The modulation of the chemical microenvironment surrounding metal nanoparticles (NPs) is an effective means to enhance the selectivity and activity of catalytic reactions. Herein, a post‐synthetic modification strategy is developed to modulate the hydrophobic microenvironment of Ru nanoparticles encapsulated in a metal–organic framework (MOF), MIP‐206, namely Ru@MIP‐F*_x_* (where *x* represents perfluoroalkyl chain lengths of 3, 5, 7, 11, and 15), in order to systematically explore the effect of the hydrophobic microenvironment on the electrocatalytic activity. The increase of perfluoroalkyl chain length can gradually enhance the hydrophobicity of the catalyst, which effectively suppresses the competitive hydrogen evolution reaction (HER). Moreover, the electrocatalytic production rate of ammonia and the corresponding Faraday efficiency display a volcano‐like pattern with increasing hydrophobicity, with Ru@MIP‐F_7_ showing the highest activity. Theoretical calculations and experiments jointly show that modification of perfluoroalkyl chains of different lengths on MIP‐206 modulates the electronic state of Ru nanoparticles and reduces the rate‐determining step for the formation of the key intermediate of N_2_H_2_^*^, leading to superior electrocatalytic performance.

## Graphical abstract

The Ru@MIP‐F_7_ catalyst is successfully synthesized by a post‐synthetic modification strategy. Strikingly, the electronic structure and hydrophobic microenvironmental modifications tailor the *d*‐band center position to optimize the adsorption of reaction intermediates, endowing the Ru@MIP‐F_7_ catalyst with superior activity in electrochemical nitrogen reduction reactions (Faraday efficiency: 40.94%, yield: 42.29 µg h^−1^ mg_cat._^−1^), far superior to the corresponding counterparts.

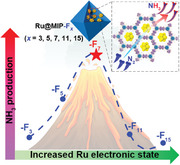

## 1 Introduction

The ambient electrochemical nitrogen reduction reaction (NRR) is an eco‐friendly and attractive alternative to the energy‐intensive Haber‐Bosch process for ammonia production.^[^ ^1^, ^2^, ^3^ ^]^ However, the unsatisfactory ammonia yield rate and Faradic efficiency (FE) of electrochemical NRR were caused by the competitive hydrogen evolution reaction (HER) and sluggish reaction kinetics, thus limiting its practical application.^[^ ^4^, ^5^, ^6^ ^]^ Currently, transition metal materials are generally regarded as promising catalysts for N_2_ chemisorption and activation, attributable to their available empty and filled *d* orbitals.^[^ ^7^, ^8^, ^9^ ^]^ Consequently, considerable efforts have been devoted to the development of high‐efficiency transition metal‐based catalysts.^[^ ^10^ ^]^ Although great progress has been made in transition metal‐based catalysts, there is still a big gap between practical applications, mainly due to the following factors: First, the robust nonpolar N≡N triple bond possesses a high bond energy of ≈941 kJ·mol^−1^, which poses substantial challenges for cleavage and hydrogenation.^[^ ^11^, ^12^, ^13^ ^]^ Second, most transition metal‐based catalysts exhibit only weak adsorption of nitrogen molecules and lack effective active sites for nitrogen activation.^[^ ^14^, ^15^, ^16^ ^]^ Third, the NRR reaction, occurring within aqueous phase systems, frequently results in most protons and electrons contributing to HER rather than NRR, owing to the strong metal‐H bonding facilitated by metal *d*‐band states proximate to the Fermi level (E_f_).^[^ ^17^, ^18^, ^19^ ^]^

To surmount these challenges, it is desirable to design a novel catalyst with a specific electronic structure and composition that inhibits HER and effectively enhances NRR performance. Metal nanoparticles (NPs) have demonstrated efficacy as electrocatalysts for nitrogen reduction.^[^ ^20^, ^21^, ^22^ ^]^ The regulation of the electronic properties of the active site, along with the microenvironment in which it is situated, is crucial for catalytic reactions.^[^ ^23^ ^]^ Modulating the surface properties of NPs typically involves the attachment of various functional molecules to their surfaces, however, this can hinder the accessibility of the metal active center and reduce the efficiency of the catalytic reaction.^[^ ^24^, ^25^, ^26^ ^]^ Therefore, constructing the ideal catalyst, which not only provides accessible metal active centers but also modulates the interaction between the catalyst and reactants, poses a significant challenge. Introducing a suitable hydrophobic microenvironment around the metal nanoparticles not only optimizes the electronic state of the metal center but also effectively enriches the hydrophobic substrate, thereby enhancing the catalytic reaction.^[^ ^27^ ^]^ Specifically, the hydrophobic microenvironment can restrict the penetration of trace water molecules in the NRR reaction, thus effectively inhibiting the competitive HER side reaction.^[^ ^28^ ^]^

Metal–organic frameworks (MOFs) are a class of crystalline porous materials with tailorable clusters, linkers, and pores that offer many advantages in catalysis.^[^ ^29^, ^30^, ^31^ ^]^ Particularly, MOFs are considered ideal candidates for precisely modulating the microenvironment of NRR catalysts owing to their tailorable and designable composition and structure.^[^ ^32^, ^33^, ^34^ ^]^ Moreover, the stable pore structure of MOFs not only supports and confines NPs but also allows the functional groups suspended on the pore walls to effectively modulate the electronic state of metal NPs, thereby enhancing the catalytic activity of the NRR reaction.^[^ ^35^ ^]^ In recent years, while most metal NPs@MOF catalysts have focused on utilizing MOFs as carriers to stabilize the metal NPs, scant attention has been paid to the influence of the chemical microenvironment around metal NPs on the catalytic performance of NRR.^[^ ^36^ ^]^ With all the above in mind, there is an urgent need to develop more effective methods for the precise regulation of the chemical microenvironment of NRR metal NPs@MOF catalysts.

**Scheme 1:**
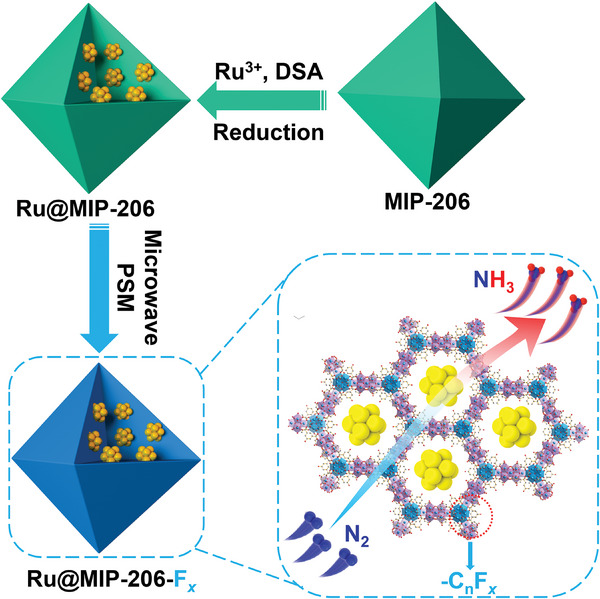
Schematic illustration showing the fabrication of Ru@MIP‐F*_x_* via postsynthetic modification for electrocatalytic N_2_ reduction.

In this work, a hydrophobic microenvironment‐enhanced NRR electrochemical system utilizing ruthenium nanoparticles (Ru NPs) embedded in a metal–organic framework, MIP‐206, termed Ru@MIP‐F*_x_* composite, is strategically developed (**Scheme** **1**). A series of post‐modified Ru@MIP‐F*_x_* (where *x* = 3, 5, 7, 11, and 15) were synthesized by grafting perfluoroalkyl acids molecules of different chain lengths on MIP‐206 using a microwave‐assisted method. The hydrophobic microenvironment of the obtained Ru@MIP‐F*_x_* gradually increased with the increase of perfluoroalkyl acid chain length, and the electrocatalytic NRR activity was gradually enhanced. Notably, the catalytic activity of Ru@MIP‐F*_x_* shows a typically volcano‐type curve, with the highest point at *x* = 7 (Faraday efficiency: 40.94%, yield: 42.29 µg h^−1^ mg_cat._^−1^), which significantly outperformed the pristine Ru@MIP‐206. This observation underscores that a moderately hydrophobic microenvironment is crucial for achieving superior NRR catalytic efficiency. Density functional theory (DFT) calculations and experimental analyses revealed that the incorporation of fluorine‐containing molecules induced a deficiency in electron density in Ru, shifting the *d*‐band center downward from the Fermi level and thereby optimizing nitrogen intermediate adsorption, enhancing NRR catalytic performance. This work highlights the impact of the hydrophobic microenvironment on NRR performance, highlighting the critical role of moderate hydrophobicity.

## 2 Results and Discussion

### 2.1 Synthesis and Characterization

**Figure 1:**
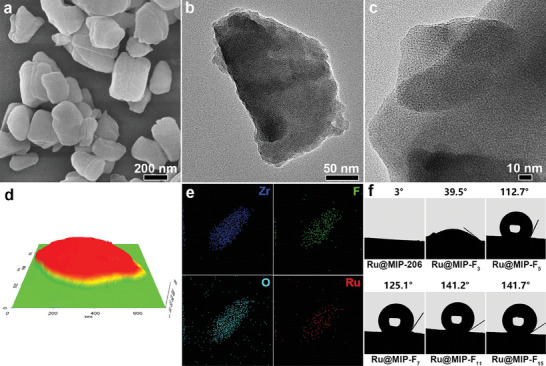
a) SEM image, b) low‐magnification, and c) high‐magnification TEM images, and d) AFM 3D topographic distribution image, and e) EDX mapping of Zr, F, O, and Ru elements of Ru@MIP‐F_7_. f) Static water contact angles of Ru@MIP‐206 and Ru@MIP‐F*_x_* (*x* = 3, 5, 7, 11, and 15).

A series of Ru@MIP‐F*_x_*, including Ru@MIP‐206, Ru@MIP‐F_3_, Ru@MIP‐F_5_, Ru@MIP‐F_7_, Ru@MIP‐F_11,_ and Ru@MIP‐F_15_, have been prepared and all exhibiting similar morphologies under scanning electron microscopy (SEM) (Figures S1 and S2, Supporting Information). Taking Ru@MIP‐F_7_ as a representative, its typical SEM image shows a uniform large sheet‐shaped with a diameter of ca. 400 nm (**Figure** **1**a). Furthermore, transmission electron microscopy (TEM) observation of Ru@MIP‐F_7_ shows that the extremely tiny Ru NPs are highly dispersed throughout the MOF nanosheets with an average size of less than 1.0 nm (Figure 1b,c; Figure S3, Supporting Information). In addition, the MIP‐206, Ru@MIP‐206, and Ru@MIP‐F*_x_* (*x* = 3, 5, 7, 11, and 15) samples also show similar structure and morphology (Figures S4–S9, Supporting Information). The thickness of the Ru@MIP‐F_7_ nanosheet catalyst was determined to be ≈200 nm by atomic force microscopy (AFM, Figure 1d; Figure S10, Supporting Information). Elemental energy‐dispersive X‐ray (EDX) mapping images further confirmed the uniform distribution of Zr, F, O, and Ru elements (Figure 1e; Figure S11, Supporting Information). The water contact angle of Ru@MIP‐206 is ≈3°, significantly increased from 39.5° to 141.7° as the length of the introduced perfluoroalkyl chains increased, demonstrating that post‐synthetic modification with perfluoroalkyl chains effectively modulates the MOF's hydrophobic microenvironment, which has a crucial impact on the electrocatalytic reaction (Figure 1f; Figure S12, Supporting Information).

The powder X‐ray diffraction (XRD) patterns of MIP‐206, Ru precursor@MIP‐206, Ru@MIP‐206, and all Ru@MIP‐F*_x_* match well with simulated MIP‐206, with good crystallinity (Figures S13 and S14, Supporting Information). The XRD characterization did not reveal any diffraction peaks of Ru nanoparticles, which indicates that the size of the metal nanoparticles is very small, in agreement with the TEM analysis. The inductively coupled plasma atomic emission spectroscopy (ICP‐AES) results indicate that the Ru contents in Ru@MIP‐F*_x_* (*x* = 3, 5, 7, 11, and 15) are similar, ranging from 1.75–2.24 wt% (Table S1, Supporting Information). The slight decrease in Ru loading after the introduction of perfluoroalkyl groups of different chain lengths could be attributed to the slight leaching of Ru nanoparticles during the modification process. Analysis of the Ru@MIP‐F*_x_* (*x* = 3, 5, 7, 11, and 15) samples by ^19^F nuclear magnetic resonance (NMR) (Figure S15, Supporting Information) can further confirm the similarity in molecular weight of the F‐containing post‐synthetic modifications. The Brunauer–Emmett–Teller (BET) surface area of MIP‐206, Ru@MIP‐206, and Ru@MIP‐F_7_ have been tested by nitrogen sorption at 77 K (Figure S16, Supporting Information). The decrease in surface area after introducing fluorine‐containing molecules is caused by part of the pore in the MOFs by the part of the Ru nanoparticles and functional groups.

### 2.2 Electrochemical NRR Performance

**Figure 2:**
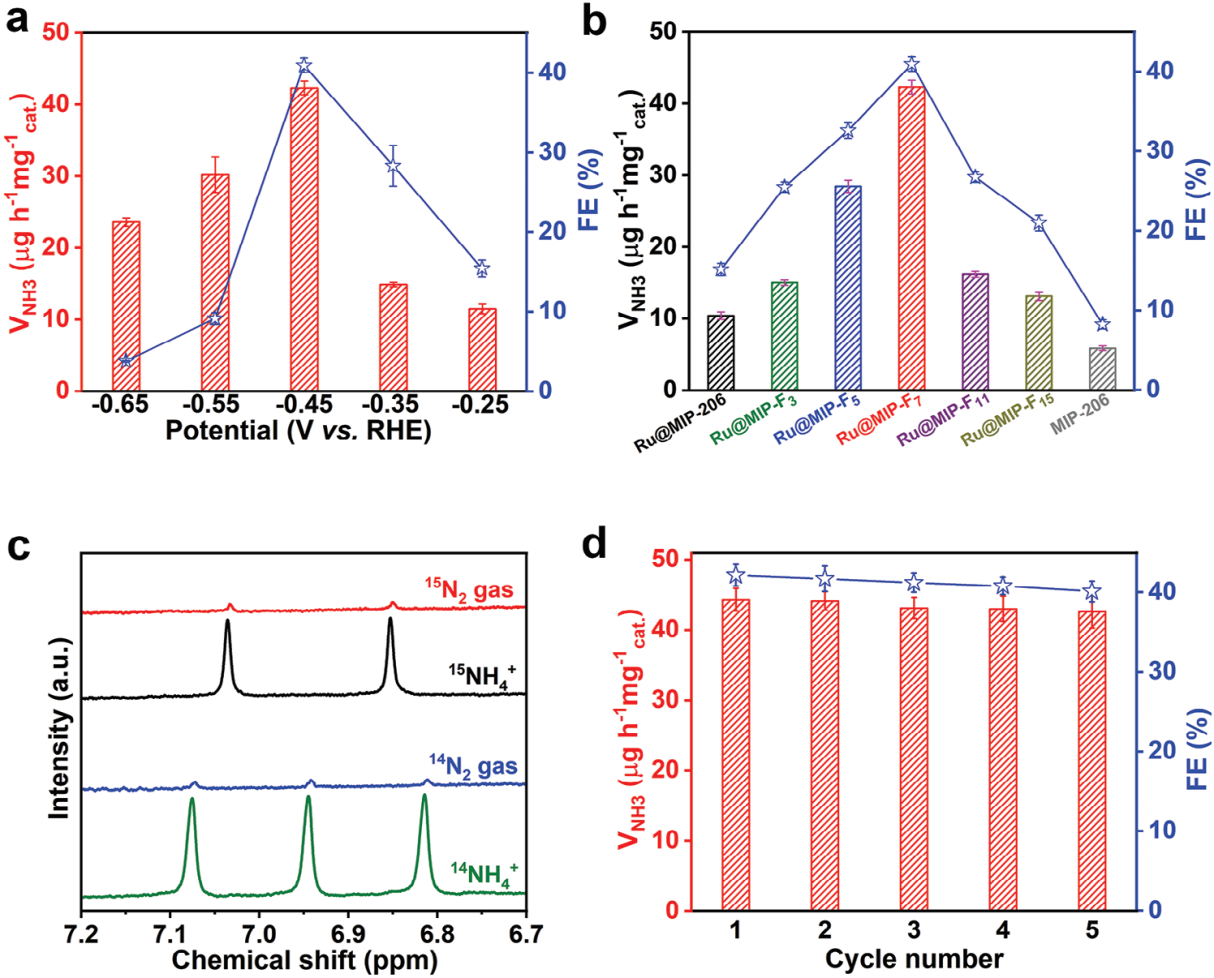
The ammonia yield rates and FE of a) Ru@MIP‐F_7_ at various potentials, and b) the control catalysts, including Ru@MIP‐206, Ru@MIP‐F_3_, Ru@MIP‐F_5_, Ru@MIP‐F_7_, Ru@MIP‐F_11_, Ru@MIP‐F_15_, and MIP‐206 at −0.45 V versus RHE. c) The ^1^H NMR spectra of the electrolyte with ^15^N_2_ and ^14^N_2_ feeding gas after electroreduction with Ru@MIP‐F_7_. d) FE and NH_3_ yield rate during the recycling test under the potential of −0.45 V versus RHE.

To explore the catalytic activity of Ru@MIP‐F_7_ in ambient nitrogen fixation, we conducted the electrochemical tests in an N_2_‐saturated 0.1 m Na_2_SO_4_ solution using an H‐shape electrochemical cell.^[^ ^37^ ^]^ The N_2_ gas supply was purified by flowing through a 0.5 m H_2_SO_4_ and 1 m KOH solution (Figure S17, Supporting Information). The indophenol blue spectrophotometric method and Watt and Chrisp method were applied to analyze the generated NH_3_ and possible by‐product of N_2_H_4_.^[^ ^38^ ^]^ The calibration curves for NH_3_ and N_2_H_4_ assay are shown in Figures S18 and S19 (Supporting Information). All potentials were measured relative to a reversible hydrogen electrode (RHE) scale. We first explored the electrocatalytic NRR performance of Ru@MIP‐F_7_ as a function of potential and examined it by linear sweep voltammetry (LSV) in a 0.1 m Na_2_SO_4_ solution saturated with Ar and N_2_ (Figure S20, Supporting Information). Notably, when the applied potential is more negative than −0.30 V, the LSV curve obtained in the N_2_‐saturated environment displayed a significant increase in current density, revealing that Ru@MIP‐F_7_ shows catalytic performance for electroreduction N_2_. Chronoamperometric curves from −0.25 to −0.65 V were collected over 7200 s and presented in Figure S21 (Supporting Information). Related UV–vis absorption spectra of the electrolytes stained with indophenol indicator are shown in Figure S22 (Supporting Information), and the highest absorbance peak appears at −0.45 V. Combined with the collected data, the FEs and the average NH_3_ yield rates of Ru@MIP‐F_7_ at different potentials are calculated. When the applied potential is −0.45 V, Ru@MIP‐F_7_ offers a maximum NH_3_ yield of 42.29 µg h^−1^ mg_cat._^−1^ and a highest FE of 40.94%, outperforming pristine Ru@MIP‐206 and pure carbon paper (CP) shown in **Figures** **2**a and S23 (Supporting Information), and superior to those of most reported metal NRR electrocatalysts in neutral environment (Table S2, Supporting Information). At the same time, the amount of NH_3_ increased with time, implying that the catalytic process was continuous (Figure S24, Supporting Information).

Significantly, the by‐product N_2_H_4_ was undetected in any of the final electrolytes at all tested potentials (Figure S25, Supporting Information), revealing that Ru@MIP‐F_7_ possesses excellent selectivity for NRR. The NH_3_ yields were also determined by ^1^H NMR quantitative spectroscopy (Figure S26, Supporting Information), and the NH_3_ yields were in general agreement with the results of the indophenol blue test. We also prepared pure MIP‐206, Ru@MIP‐206, and Ru@MIP‐F*_x_* (*x* = 3, 5, 7, 11, and 15) catalysts with different chain lengths of perfluoroalkyl acids and evaluated for their NRR activity. As shown in Figures 2b and S27 (Supporting Information), Ru@MIP‐F_3_ displays a slightly higher NH_3_ yield of 15.03 µg h^−1^ mg_cat._^−1^ than that of Ru@MIP‐206 (10.41 µg h^−1^ mg_cat._^−1^). When chain lengths of perfluoroalkyl acids are increased to F_7_, the NH_3_ yield is significantly enhanced to 42.29 µg h^−1^ mg_cat._^−1^, and then NH_3_ yield is decreased to 13.12 µg h^−1^ mg_cat._^−1^ as chain lengths of perfluoroalkyl acids is increased F_15_. It suggests that the NRR activity of Ru@MIP‐F*_x_* is directly related to the chain length of perfluoroalkyl acids, and the Ru@MIP‐F_7_ sample exhibits the best performance. At a potential of −0.45 V, none of the above catalysts detected the by‐product N_2_H_4_ (Figure S28, Supporting Information). A comparison of time‐dependent current density curves (Figure S29, Supporting Information) for different catalysts shows that Ru@MIP‐F_7_ possessed the highest current density, revealing that it may have better activity. Additionally, we also compared the LSV curves of different catalysts in both Ar and N_2_‐saturated electrolytes (Figures S30 and S31, Supporting Information) and revealed that as the length of the perfluoroalkyl acids chain increases, the limiting current has different degrees of reduction, indicating that the modification of the hydrophobic microenvironment significantly mitigated HER activity in these catalysts. Furthermore, we found that Ru@MIP‐F_7_ catalyst has the largest NRR current between −0.3 and −0.6 V, suggesting that a moderate level of hydrophobicity is crucial for effective nitrogen reduction. Generally, NRR and HER are competitive processes, with NRR involving the adsorption and gradual hydrogenation of N_2_ molecules, and this process requires the participation of protons.^[^ ^39^ ^]^ The hydrophobicity is too weak and has no inhibitory effect on the HER performance, while the hydrophobicity is too strong, which not only inhibits the HER but also has a great inhibitory effect on the reduction of nitrogen.

To confirm that ammonia is generated by NRR on Ru@MIP‐F_7_ catalyst, two control experiments were carried out in N_2_‐saturated solutions at open‐circuit potentials and in Ar‐saturated solutions at −0.45 V, respectively. As expected, these experiments only offer blank results (Figure S32, Supporting Information). All these results testify that the detected NH_3_ was produced via electrochemical conversion of N_2_ over Ru@MIP‐F_7_. Furthermore, isotopic labeling experiments were performed to verify the origin of the NH_3_. As observed in the ^1^H NMR spectra (Figure 2c), the standard samples show a doublet for ^15^NH_4_^+^ and a triplet for ^14^NH_4_^+^. When ^15^N_2_ was used as the feed gas, only a doublet was detected.^[^ ^40^ ^]^ Therefore, the ^1^H NMR results provide strong support that the NH_3_ is absolutely identified as the production from NRR. To elucidate the electronic properties at the electrode‐solution interface, electrochemical impedance spectroscopy (EIS) was employed, revealing the electrode dynamics of the NRR process. The EIS profiles showed a smaller semicircle radius for Ru@MIP‐F_7_ compared to Ru@MIP‐206 (Figure S33, Supporting Information), indicating enhanced electron transfer rates and more rapid catalytic kinetics for Ru@MIP‐F_7_.^[^ ^41^ ^]^ To further understand the superior NRR performance of Ru@MIP‐F_7_, we measured the electrochemical double‐layer capacitance (C_dl_) of Ru@MIP‐F_7_ and Ru@MIP‐206. As shown in Figures S34 and S35 (Supporting Information), the C_dl_ of Ru@MIP‐F_7_ is significantly larger than that of Ru@MIP‐206, which implies that Ru@MIP‐F_7_ has a larger specific surface area, and thus it can be said to have more exposed catalytically active sites for NRR reactions.^[^ ^42^ ^]^ The stability of catalysts is one of the critical parameters for catalytic performance evaluation. Consecutive five recycling experiments were used to assess the stability of Ru@MIP‐F_7_ at −0.45 V. As shown in Figure 2d, Ru@MIP‐F_7_ displays a slight change in both NH_3_ yields and FE during the recycling experiment, implying that Ru@MIP‐F_7_ is rather stable for NRR. No significant change in current density was observed after 50 h of NRR electrolysis (Figure S36, Supporting Information), demonstrating the excellent long‐term electrochemical stability of Ru@MIP‐F_7_. After long‐term NRR electrolysis, XRD analysis confirmed that the catalyst remained Ru@MIP‐F_7_ in nature after NRR. Additionally, the SEM, TEM, and elemental mapping images (Figure S37, Supporting Information) of Ru@MIP‐F_7_ after long‐term electrocatalysis show negligible morphological changes. The results of the above data indicate that the Ru@MIP‐F_7_ catalyst has good NRR catalytic stability.

### 2.3 Reaction Mechanism

**Figure 3:**
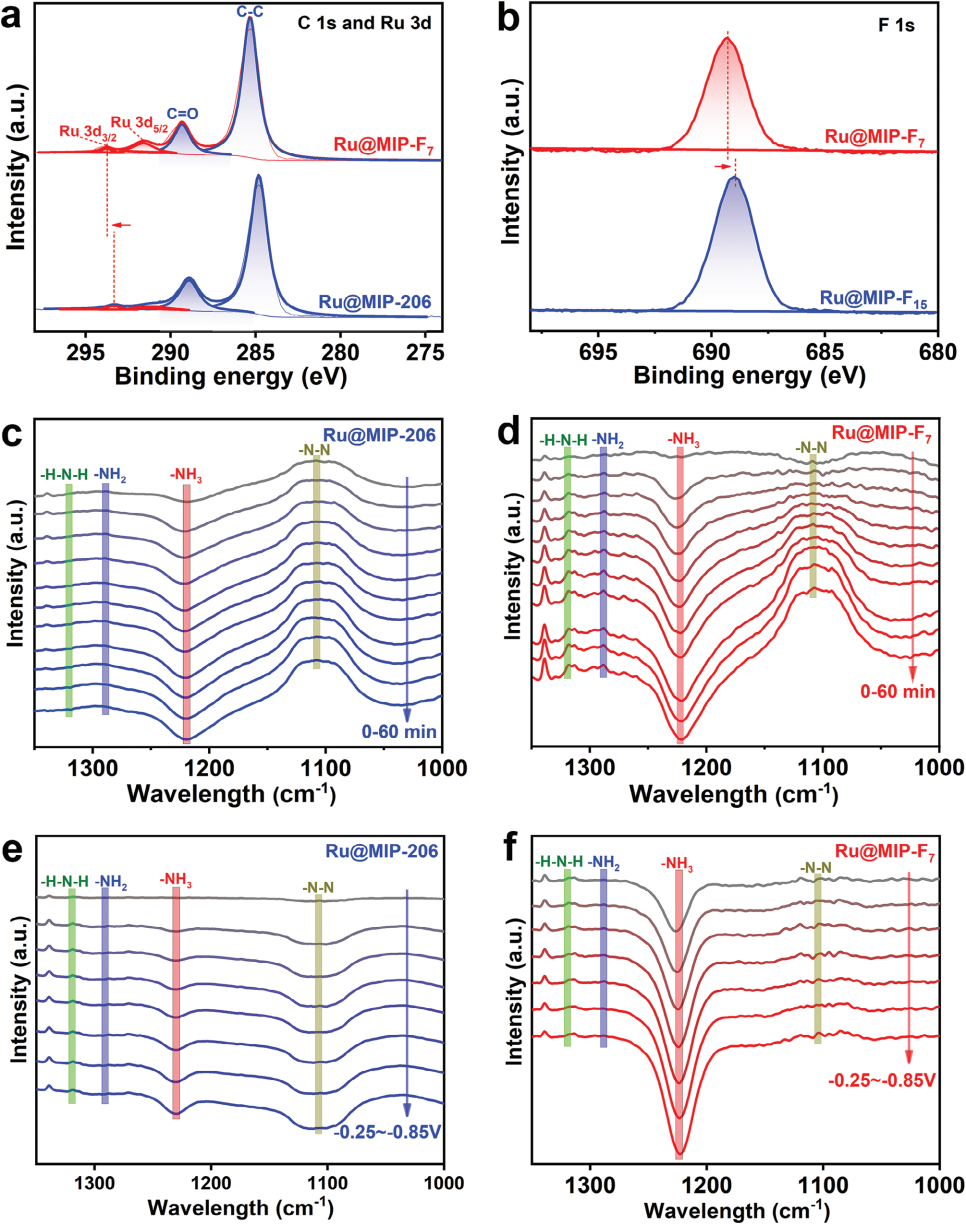
The high‐resolution XPS of a) C 1s and Ru 3d in the Ru@MIP‐206 and Ru@MIP‐F_7_, and b) F 1s in the Ru@MIP‐F_15_ and Ru@MIP‐F_7_. In situ FTIR spectra of c) Ru@MIP‐206, and d) Ru@MIP‐F_7_ during the electrocatalytic reaction at −0.45 V versus RHE for 60 min. In situ FTIR spectra of e) Ru@MIP‐206, and f) Ru@MIP‐F_7_, under different applied potentials with nitrogen as the feed gas.

To elucidate the reasons behind the high activity of the Ru@MIP‐F_7_ catalyst, we employed X‐ray photoelectron spectroscopy (XPS) to characterize its electronic state. The XPS survey spectrum confirms the presence of Ru, Zr, and F elements in the Ru@MIP‐F_7_ catalyst (Figure S38, Supporting Information). As shown in **Figures** **3**a and S39 (Supporting Information), the binding energies at 291.4 and 293.3 eV are attributed to Ru 3d_5/2_ and Ru 3d_3/2_ of the Ru@MIP‐206, while the peaks at 284.8 and 288.9 eV correspond to the C─C and C═O bonds, respectively.^[^ ^43^ ^]^ Compared with Ru@MIP‐206 catalyst, Ru@MIP‐F_7_ catalyst was significantly shifted towards higher binding energies, suggesting electron deficiency at the metal Ru sites due to the incorporation of perfluorobutyric acid molecules. Similarly, the Ru 3p high‐resolution spectrum demonstrates a similar trend (Figure S40, Supporting Information).^[^ ^44^ ^]^ Additionally, the F 1s peak in the Ru@MIP‐F_7_ catalyst showed a shift toward lower binding energy compared to the Ru@MIP‐F_15_ catalyst, indicating electron gain by F from Ru, enhanced with the lengthening of the perfluoroalkyl chains (Figure 3b).^[^ ^45^ ^]^ The above results indicate that the introduction of perfluoroalkyl molecules not only modulate the hydrophobic microenvironment of the catalyst but also optimize the electronic structure of the Ru metal sites, thereby enhancing NRR activity.

To compare the NRR catalytic performance between Ru@MIP‐206 and Ru@MIP‐F_7_ catalysts, in situ Fourier transform infrared (FTIR) spectroscopy was performed. Figure 3c,d displays the in situ FTIR spectra captured from the surfaces of Ru@MIP‐206 and Ru@MIP‐F_7_ electrodes during continuous electrolysis for 60 min at −0.45 V versus RHE in a N_2_‐saturated electrolyte solution. The gradual intensification of absorption bands, which are attributable to the ‐N_2_H_y_ intermediate, indicates efficient adsorption and activation of N_2_ on the Ru@MIP‐F_7_ surface. Specifically, the observed absorption bands ranging from 1000 to 1400 cm^−1^ correspond to the N─N stretching (1108 cm^−1^), adsorbed ‐NH_3_ (1220 cm^−1^), ‐NH_2_ wobbling (1288 cm^−1^) and H─N─H bending (1318 cm^−1^) models of the ‐N_2_H_y_ intermediates, respectively.^[^ ^46^, ^47^, ^48^, ^49^ ^]^ The in situ FTIR spectra can confirm that the Ru@MIP‐206 and Ru@MIP‐F_7_ electrode surfaces follow an associative mechanism.^[^ ^50^ ^]^ Over time, the Ru@MIP‐F_7_ catalyst exhibited stronger characteristic absorption peaks than Ru@MIP‐206, confirming its superior catalytic activity. Similarly, the in situ FTIR spectra of Ru@MIP‐206 and Ru@MIP‐F_7_ catalysts (Figure 3e,f) at different applied potentials with nitrogen as the feedstock gas demonstrate a similar trend. The above results further demonstrate that the introduction of perfluoroalkyl molecules on MOFs modulates the hydrophobic microenvironment around the metal active sites, which is conducive to the enhancement of NRR catalytic performance.

**Figure 4:**
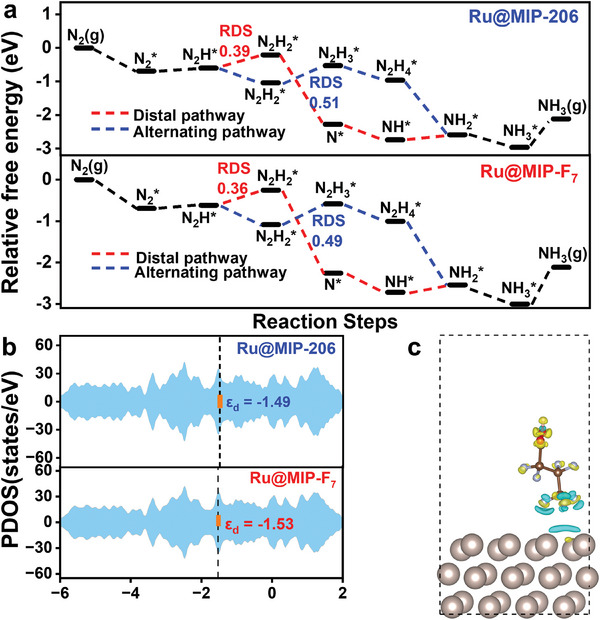
a) The energy variations of NRR process along reaction path of Ru@MIP‐206 and Ru@MIP‐F_7_. b) The partial density of states and d‐band center values of Ru atoms on Ru@MIP‐206 and Ru@MIP‐F_7_. c) The electron density difference plot of Ru@MIP‐F_7_ before and after heptafluorobutyric acid introduce in ± 0.0005 e/Bohr^3^ isosurface.

Density functional theory (DFT) calculation was performed to deeply understand the reactivity trends between Ru nanoparticles and their introduced perfluorobutyric acid/perfluorooctanoic acid (F_7_/F_15_)‐contained counterparts. The Gibbs free energy (ΔG) variation across the reaction pathways for Ru@MIP‐206 and Ru@MIP‐F_7_ was analyzed, indicating that the protonation of N_2_H^*^ and N_2_H_2_^*^ is the rate‐determining step (RDS) for distal pathway and alternating pathway respectively. Ru@MIP‐F_7_ consistently displayed a lower energy barrier of RDS than that of Ru@MIP‐206 and Ru@MIP‐F_15_ (**Figure** **4**a; Figure S41, Supporting Information). Although the final desorption of NH_3_^*^ is endothermic, protonation of NH_3_^*^ is considered a favorable step.^[^ ^51^ ^]^ The abovementioned results fit the experimental superior NRR activity of Ru@MIP‐F_7_ to the talked Ru counterparts. To reveal the interesting NRR activity trends, the *d*‐band center (ε_d_) of Ru before and after the F_7_/F_15_ modification has been investigated. Shifting the *d*‐band center upward towards the Fermi level tends to strengthen electron aggregation, resulting in stronger interactions with reactants, conversely, a downward shift has the opposite effect. The ε_d_ value of Ru@MIP‐F_7_ (−1.53 eV) sits between that of Ru@MIP‐206 (−1.49 eV) and Ru@MIP‐F_15_ (−1.57 eV) (Figures 4b and S42, Supporting Information), indicating that Ru@MIP‐F_7_’s *d*‐band is ideally positioned for optimal catalytic activity. Given that appropriate binding between the substrate and active center/catalyst is favorable, and too strong strength is detrimental to catalytic reactions, based on the Sabatier principle,^[^ ^52^ ^]^ these results well explain and should account for the order of experimental activity. Bader charge analysis and electron density difference analysis (Figure 4c) reveal that the downshifts of the *d*‐band center with the increase of fluorine chain length is attributed to the electron exchanging from Ru metals to F_7_/F_15_ molecule with the values of 0.13 and 0.22 eV, which is consistent with the above XPS results.

## 3 Conclusion

In summary, a zirconium‐based MOF, MIP‐206, with modulated hydrophobic microenvironments, was successfully engineered to enhance the catalytic activity for NRR. By grafting perfluoroalkyl acids of varying lengths, the hydrophobicity of the catalyst was effectively modulated, leading to a suppression of competitive HER and improved nitrogen adsorption. The electrocatalytic production of NH_3_ follows an interesting volcano‐type trend with increased hydrophobicity in the MOF. The Ru@MIP‐F_7_, modified with perfluorobutyric acid, exhibited the highest electrocatalytic performance, suggesting that proper modulation of the hydrophobic microenvironment is crucial for enhancing NRR performance. DFT analysis indicates that the rate‐determining step for Ru@MIP‐F_7_ towards NRR has a substantially lower energy barrier compared to Ru@MIP‐206 and Ru@MIP‐F_15_, providing a theoretical explanation for the superior catalytic activity of Ru@MIP‐F_7_. This research underscores the importance of hydrophobic microenvironment modulation in MOFs for optimizing NRR catalytic activity and offers valuable guidance for the development of future catalysts.

## Conflict of Interest

The authors declare no conflict of interest.

The data that support the findings of this study are available from the corresponding author upon reasonable request.

## Supporting information

Supporting Information
