# Supplementary material for: Hydrophobic Microenvironment Modulation of Ru Nanoparticles in Metal–Organic Frameworks for Enhanced Electrocatalytic N2 Reduction
Source: Adv Sci (Weinh). 2024 Jul 10;11(34):2405210. doi: 10.1002/advs.202405210 (PMC11425667; doi:10.1002/advs.202405210)
Supplement: Supplementary file 1 — Supporting Information [file ADVS-11-2405210-s001.docx]

**Supporting Information**

**Hydrophobic Microenvironment Modulation of Ru Nanoparticles in Metal–Organic Frameworks for Enhanced Electrocatalytic N_2_ Reduction**

*Lulu Wen****†****, Xiaoshuo Liu****†****, Xinyang Li, Hanlin Zhang, Shichuan Zhong, Pan Zeng, Syed Shoaib Ahmad Shah, Xiaoye Hu, Weiping Cai, and Yue Li**

Dr. L. Wen, X. Li, H. Zhang, S. Zhong, P. Zeng, Prof. X. Hu, Prof. W. Cai, Prof. Y. Li

Key Lab of Materials Physics, Anhui Key Lab of Nanomaterials and Nanotechnology, Institute of Solid State Physics, Hefei Institutes of Physical Science, Chinese Academy of Sciences, Hefei 230031, Anhui, P. R. China.

Dr. X. Liu

Key Laboratory of Energy Thermal Conversion and Control of Ministry of Education, School of Energy and Environment, Southeast University, Nanjing 210096, P. R. China

Prof. Y. Li

School of Physical Science and Technology, Tiangong University, Tianjin, 300387, P. R. China

H. Zhang, P. Zeng

University of Science and Technology of China, Hefei 230026, P. R. China

Dr. S. S. A. Shah

Department of Chemistry, School of Natural Sciences, National University of Sciences and Technology, Islamabad 44000, Pakistan

*To whom correspondence should be addressed.

E-mail: yueli@issp.ac.cn

**S1. Materials and Instrumentation**

All of the chemicals were commercially sources and directly utilized: zirconium (IV) chloride (ZrCl_4_, Aladdin Industrial Inc., 98%), sodium hypochlorite (NaClO, Sinopharm Chemical Reagent Co., Ltd., CP), isophthalic acid (IPA, Alfa Aesar), sodium nitroferricyanide dehydrate (Aladdin Industrial Corporation, AR), formic acid (Sinopharm Chemical Reagent Co., Ltd., AR), sodium hydroxide (NaOH, Sinopharm Chemical Reagent Co., Ltd., AR), acetone (Sinopharm Chemical Reagent Co., Ltd., AR), 117 Nafion membrane (Dupont), trifluoroacetic acid (Energy Chemical, 98%), Ruthenium chloride hydrate (RuCl_3_·*x*H_2_O, Aladdin Industrial Inc.), perfluoropropionic acid (Energy Chemical, 98%), acetonitrile (Sinopharm Chemical Reagent Co., Ltd., AR), sodium citrate (Sinopharm Chemical Reagent Co., Ltd., AR), heptafluorobutyric acid (Energy Chemical, 98%), Hexane (Sinopharm Chemical Reagent Co., Ltd., AR), ammonium chloride with the 15N enrichment of 99% (Aladdin Industrial Corporation, 98%), undecafluorohexanoic acid (Energy Chemical, 98%), N,N-dimethylformamide (DMF, Sinopharm Chemical Reagent Co., Ltd., AR), perfluorooctanoic acid (Sigma-Aldrich, 96%), 4-dimethylaminobenzaldehyde (Energy

Chemical, 99%), Sodium sulfate (Na_2_SO_4_, Sinopharm Chemical Reagent Co., Ltd., AR).

X-ray diffraction (XRD) patterns of the catalysts were collected using a Japan Rigaku Miniflex 600 rotation anode diffractometer with Cu Kα radiation of wavelength 1.54 Å. UV-vis absorption spectra were acquired on a Shimadzu UV-2700 spectrophotometer. X-ray photoelectron spectroscopy (XPS) are collected on an ESCALAB 250 high-performance electron spectrometer (Thermo Fisher Scientific) with monochromatized Al Ka (*hν* = 1486.6 eV). The scanning electron microscopy (SEM) images were collected on Zeiss Supra 40 scanning electron microscope at an accelerating voltage of 20 kV. ^1^H nuclear magnetic resonance (NMR) spectra were collected on a Bruker AC-400 FT (400 MHz) and DMSO-d_6_ was used as an internal to calibrate the chemical shifts in the spectra. Transmission electron microscope (TEM) and elemental mapping were taken on a JEM-2010 electron microscope at an accelerating voltage of 100 kV. Inductively coupled plasma atomic emission spectrometer (ICP-AES) tests were performed by an Optima 7300 DV equipment. The specific surface area (SBET) measurements were carried out using N_2_ adsorption/desorption at 77 K on a Micromeritics ASAP 2020 instrument.

**S2. Experimental Section**

**2.1 Preparation of catalysts.**

**Preparation of MIP-206:** MIP-206 was synthesized with minor modifications from previously established methods.^[S1]^ In a typical solvothermal synthesis, isophthalic acid (IPA, 1.1 g, 6.6 mmol) was dissolved in 5 mL of formic acid (FA) under mechanical stirring to achieve a clear homogeneous solution at ambient temperature. Subsequently, ZrCl_4_ (2 g, 8.6 mmol) was incrementally added to the solution. After stirring for 10 minutes at room temperature, the mixture was transferred to a 23 mL Teflon-lined autoclave and heated at 180 °C for 24 hours. The resultant solid was isolated by filtration, washed alternately with methanol and acetone, and air-dried overnight.

**Preparation of Ru@MIP-206:** Typically, 100 mg of activated MIP-206 sample was dispersed in 20 mL of *n*-hexane and the mixture was sonicated for 30 min.^[S2]^ Subsequently, RuCl_3_·*x*H_2_O (4.0 mg) aqueous solution was gradually introduced into the suspension under vigorous stirring. Then the resultant mixture was sonicated for another 3 hours. The harvested sample was further dried followed by treating in a stream of 20% H_2_/Ar (40 mL min^−1^) at 200 °C for 4 h to yield Ru@MIP-206.

**Preparation of** **Ru@MIP-F*_x_*:** The Ru@MIP-F*_x_* were synthesized according to the previous work with modifications.^[S3]^ Typically, 0.24 mmol perfluoroalkyl acid, 60 mg of Ru@MIP-206 and 2.4 mL acetonitrile were mixed in a 10 mL microwave vial and reacted at 60 ^o^C for 24 hours by microwave treatment. After the autoclave cooled down naturally to room temperature, the resulting MIP-206 was taken out and washed with hot acetonitrile, acetone several times alternatively, then activated by soaking in acetone for 24 hours, finally drying in air.

**2.2 Working electrode preparation:** The Ru@MIP-F*_x_* working electrode was prepared as follows. 5 mg catalyst and 20 µL Nafion solution (5 wt%) were dispersed in 480 µL ethanol/water (V : V = 3 : 1) by sonicating for 1 h to form a homogeneous ink. The dispersion of 20 µL homogeneous ink was then loaded onto a carbon paper electrode with geometric surface area of 1 cm^2^ and dried under ambient conditions.

**2.3 Electrochemical NRR measurements:** Electrochemical measurements were carried out with an H-type cell separated by a Nafion 115 membrane at room temperature and atmospheric pressure. Ru@MIP-F*_x_* was used as the working electrode, while graphite rod and Ag/AgCl were used as the counter and reference electrodes, respectively. The N_2_ electrochemical reduction was conducted in N_2_-saturated 0.1 M Na_2_SO_4_ solution at room temperature under atmospheric pressure. After N_2_ was purged into the Na_2_SO_4_ solution for at least 30 min to remove residual air, controlled potential electrolysis was performed at applied potentials for 2 h. All potentials in this study were measured against the Ag/AgCl reference electrode and converted to the RHE reference scale by *E* (*vs.* RHE) = *E* (*vs.* Ag/AgCl) + 0.197 + 0.059 × pH.

**2.4 Detection of ammonia:** The concentrations of NH_3_ produced in 0.1 M Na_2_SO_4_ solutions were determined via a widely used colorimetric method using indophenol blue method.^[S4]^ In detail, 2 mL of the reaction solution was first pipetted from the post-electrolysis electrolyte. Afterwards, the reaction solution was mixed with 2 mL of a 1 M NaOH solution containing salicylic acid and sodium citrate, 1 mL of 0.05 M NaClO and 200 µL of 1% C_5_FeN_6_Na_2_O·2H_2_O. The mixture was gently agitated for 30 s and was subsequently allowed to stand for 2 h to ensure complete color development. The UV-visible spectrum was performed to measure the absorbance of the mixture at ~655 nm. The concentration-absorbance curves were calibrated using standard NH_4_^+^ solution with a series of concentrations in the Na_2_SO_4_ electrolyte. The fitting curve (y = 0.509*x* + 0.025, R^2^ = 0.999) shows excellent linear relationship between absorbance and NH_3_ concentration in three independent calibrations.

**2.5 Determination of hydrazine:** The N_2_H_4_ present in the electrolyte was determined by the method of Watt and Chrisp.^[S5]^ The mixture of *p*-C_9_H_11_NO (5.99 g), HCl (concentrated, 30 mL), and C_2_H_5_OH (300 mL) was used as a color reagent. In detail, 3 mL electrolyte was removed from the electrochemical reaction vessel, and added into 3 mL above prepared color reagent for 15 min at room temperature. Moreover, the absorbance of the resulting solution was measured at a wavelength of 455 nm. The concentration absorbance curves were calibrated using standard N_2_H_4_ solution with a series of concentrations. The fitting curve (y = 0.742*x* + 0.043, R^2^ = 0.999) shows good linear relation of absorbance value with N_2_H_4_ concentration.

**2.6 Determination of NH_3_ yield and FE:** NH_3_ yield was calculated using the following equation:

NH_3_ yield = [NH_3_] × V / ( t × m_cat._)

FE was calculated according to following equation:

FE = 3*F* × [NH_3_] × *V* / 17 × *Q*

where [NH_3_] is the measured NH_3_ concentration; t is the potential applied time; V is the volume of the cathodic reaction electrolyte; m_cat._ is the loaded quality of catalyst; *F* is the Faraday constant and *Q* is the quantity of applied electricity.

**2.7 Isotope labeling experiment:**

To confirm that the ammonia and hydrazine detected in the catalytic experiments originated from dissolved N_2_, further experiments were performed using ^14^N_2_ and ^15^N_2_ the feeding gas.^[S6]^ Before the NRR process, the ^15^N_2_ and ^14^N_2_ were also purified with a gas-washing bottle filled with 1M KOH. After electrolysis at −0.45 V vs RHE in 0.1 M Na_2_SO_4_ solution, the post electrolyte solution was concentrated to 2.0 mL. Then, the obtained NH_4_^+^ contained solution was detected by using ^1^H NMR measurement Bruker AC-400 FT (400 MHz).

**2.8** **Calculation method:**

Spin–polarization DFT method including DFT-D3 van der Waals corrections were conducted using the Perdew–Burke–Ernzerhof (PBE) method and projector augmented wave (PAW) basis set, as implemented in the Vienna ab initio simulation package (VASP 5.4.1).^[S7-S9]^ The cutoff of 450 eV was adopted for the plane–wave basis. Convergence thresholds for the energy and atomic force were chosen as 10^-5^ eV and 0.05 eV/Å, respectively. In addition, Gamma point k-mesh grid was selected to make geometric structure optimization and vibrational frequency calculations. The Gibbs free energy variation (ΔG) for N_2_ reduction along the reaction pathway at room temperature was calculated based on the standard hydrogen electrode (SHE) model with the assistance of Vaspkit code,^[S10],[S11]^ and ΔG can be obtained by the following equation:

ΔG = ΔE + ΔZPE − TΔS + ΔG_U_ + ΔG_pH_

Herein, ΔE is the electronic energy change in ground-state, ΔZPE is the difference of zero-point energy, T is the temperature (T=298.15 K), ΔS is the entropy change; ΔG_U_ is the contribution of applied electrode potential to Gibbs free energy (ΔG_U_=0); ΔG_pH_ is the donation of H^+^ concentration to Gibbs free energy, which is calculated from ΔG_pH_ = 2.303 × *k*_B_T × pH (pH=0, ΔG_pH_=0).

To simulate the active site of metal nanoparticles, the (101) facet of Ru was used,^[S12]^ with 4 ×4 size in three layers by restricting the bottom two-layer atoms and relaxing top layer atoms. To identify the effect of perfluorobutyric acid / perfluorooctanoic acid (F_7_/F_15_) on the NRR, the single F_7_ or F_15_ molecule was introduced into the Ru (101) surface to model the Ru@MIP-F_7_ as well as the Ru@MIP-F_15_, respectively, which was shown in Figure S43.^[S13]^ A 15 Å vacuum layer was put onto the reaction surface to weaken boundary effect.


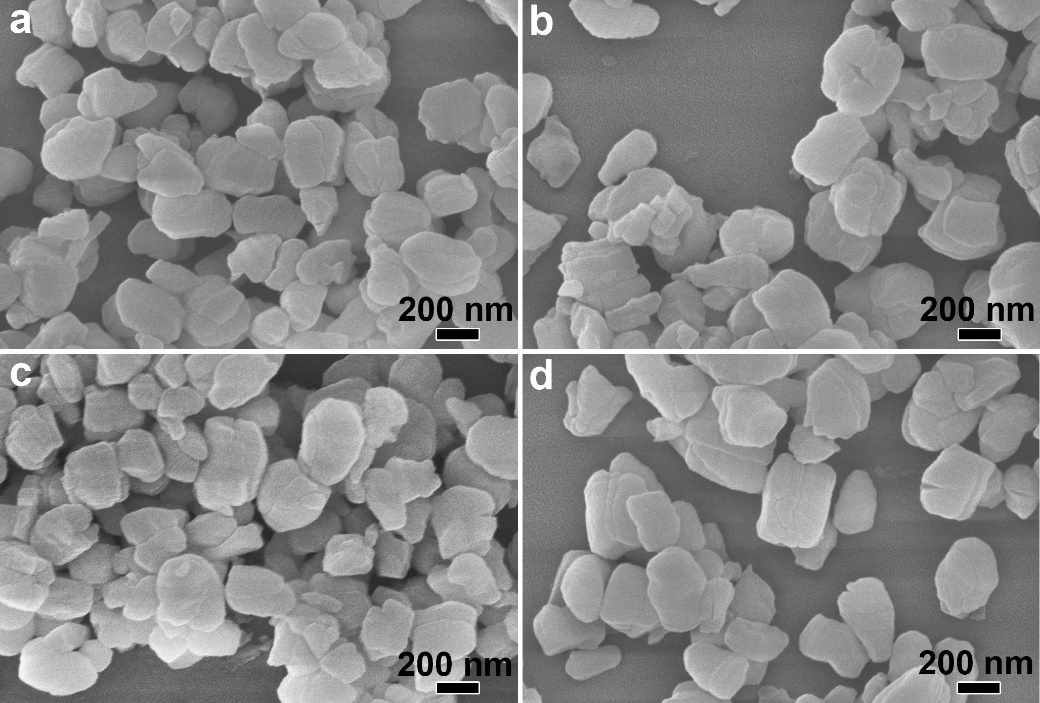


**Figure S1.** SEM images of as-synthesized (a) MIP-206, (b) Ru precursor@MIP-206, (c) Ru@MIP-206, (d) Ru@MIP-F_7_ catalysts.


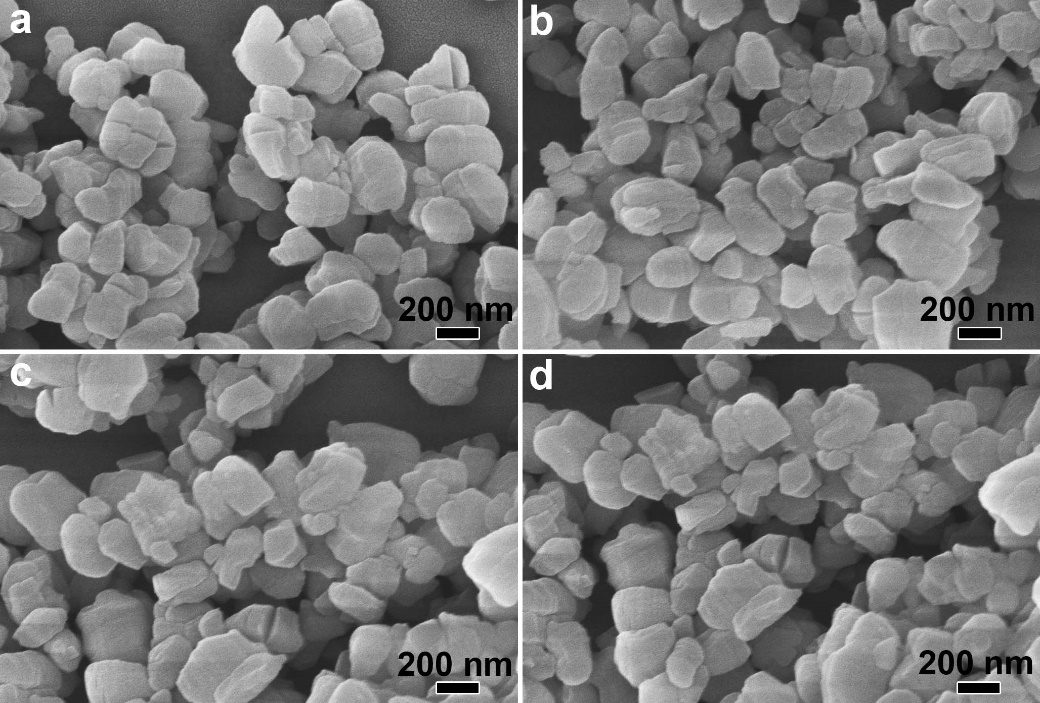


**Figure S2.** SEM images for (a) Ru@MIP-F_3_, (b) Ru@MIP-F_5_, (c) Ru@MIP-F_11_, (d) Ru@MIP-F_15_ catalysts.


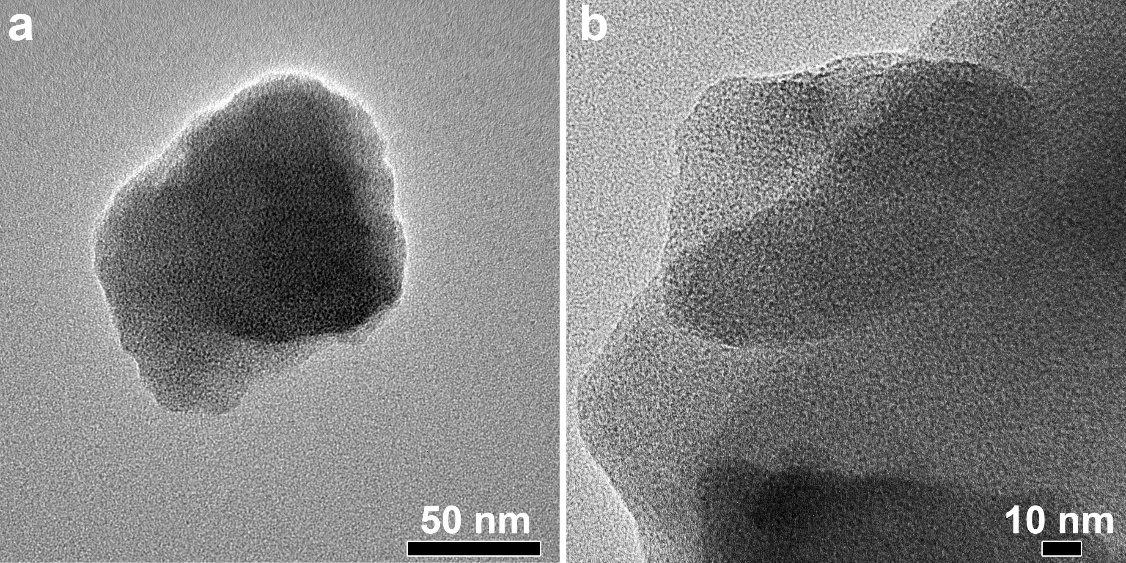


**Figure S3.** (a) Low- and (b) high-magnification TEM images of Ru@MIP-F_7_.


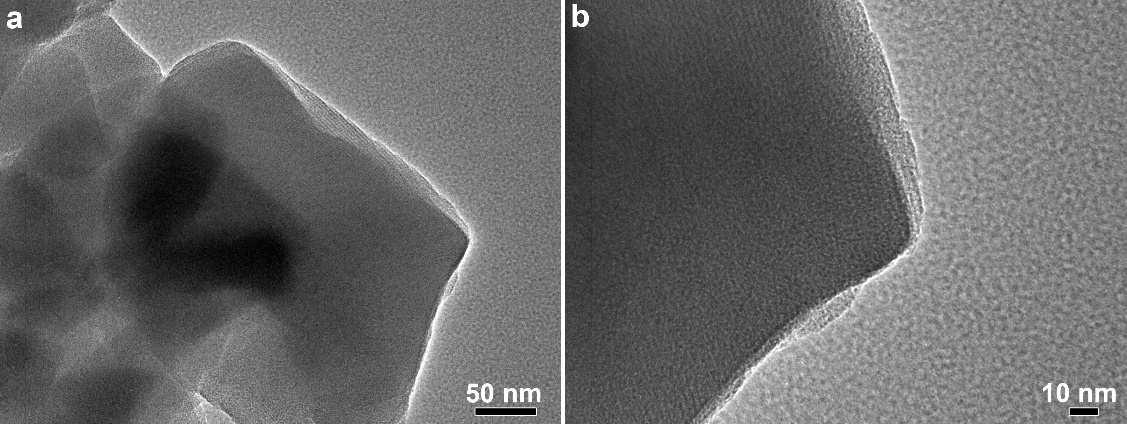


**Figure S4.** (a) Low- and (b) high-magnification TEM images of MIP-206.


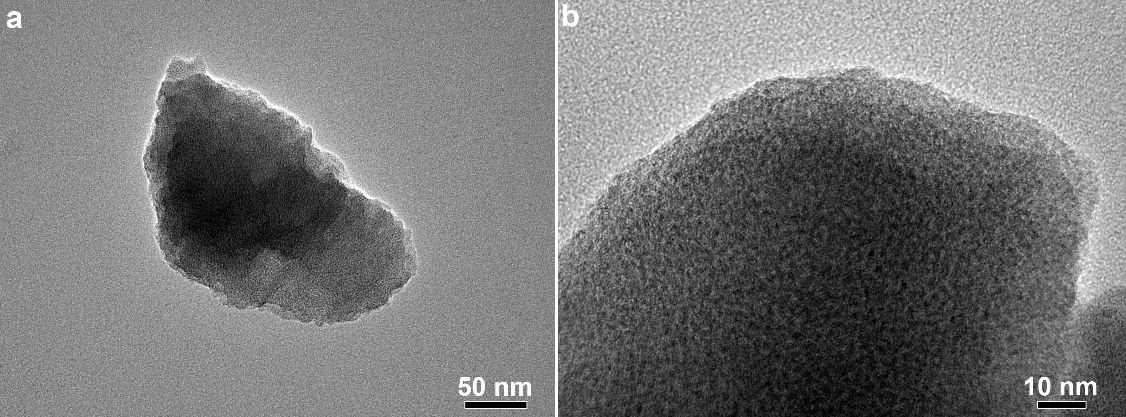


**Figure S5.** (a) Low- and (b) high-magnification TEM images of Ru@MIP-206.


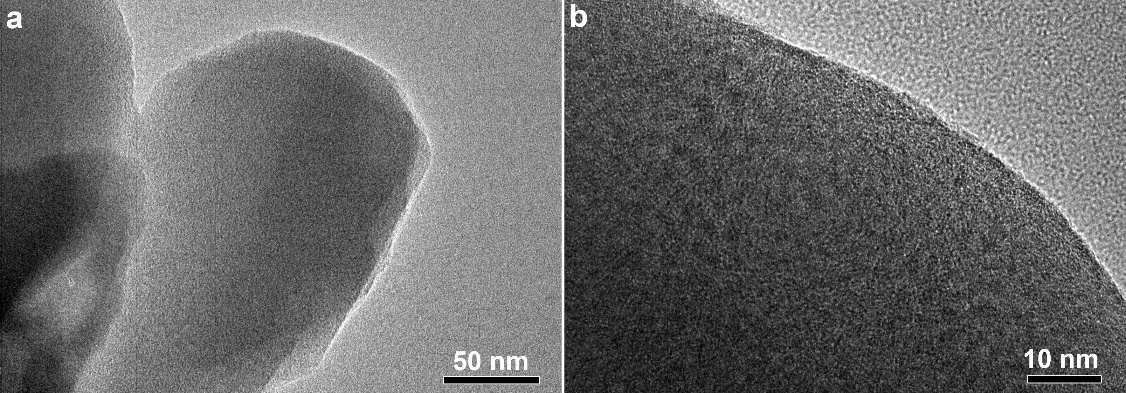


**Figure S6.** (a) Low- and (b) high-magnification TEM images of Ru@MIP-F_3_.


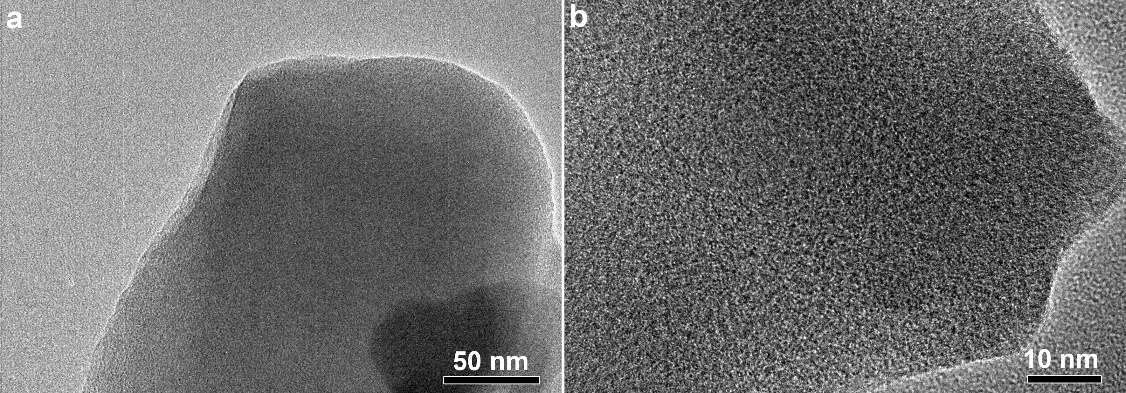


**Figure S7.** (a) Low- and (b) high-magnification TEM images of Ru@MIP-F_5_.


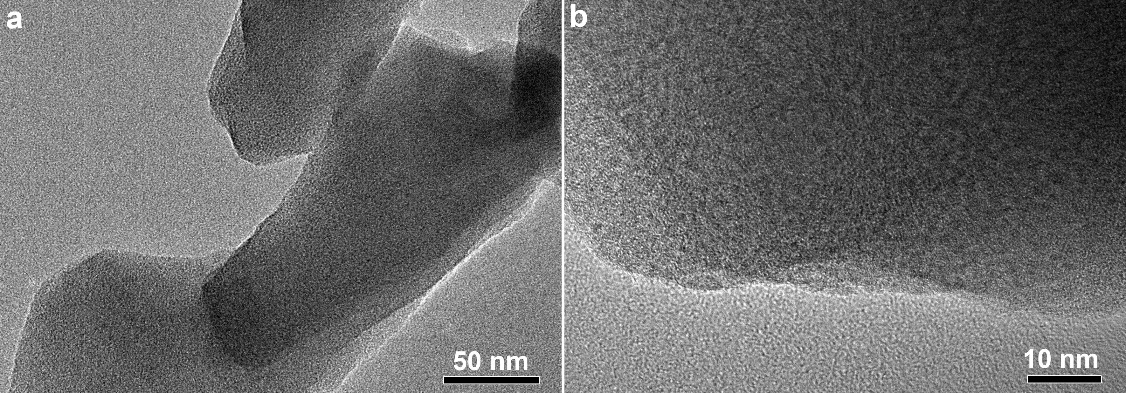


**Figure S8.** (a) Low- and (b) high-magnification TEM images of Ru@MIP-F_11_.


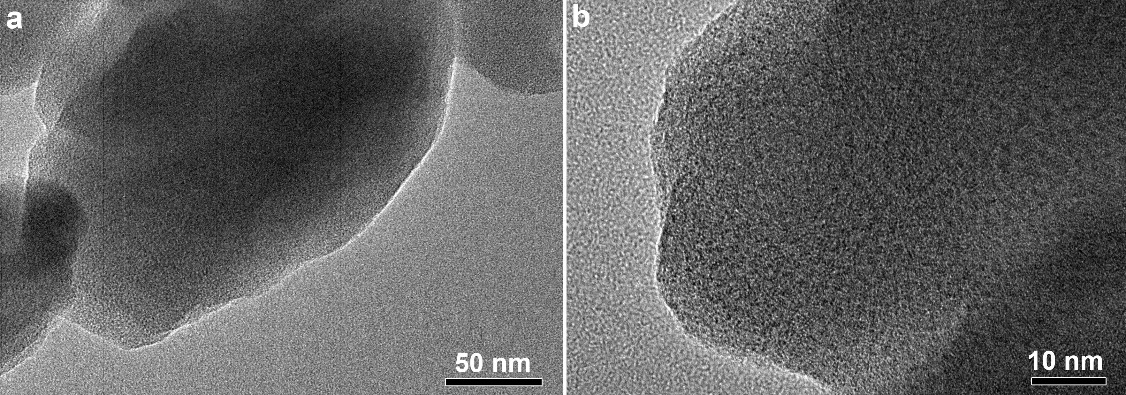


**Figure S9.** (a) Low- and (b) high-magnification TEM images of Ru@MIP-F_15_.


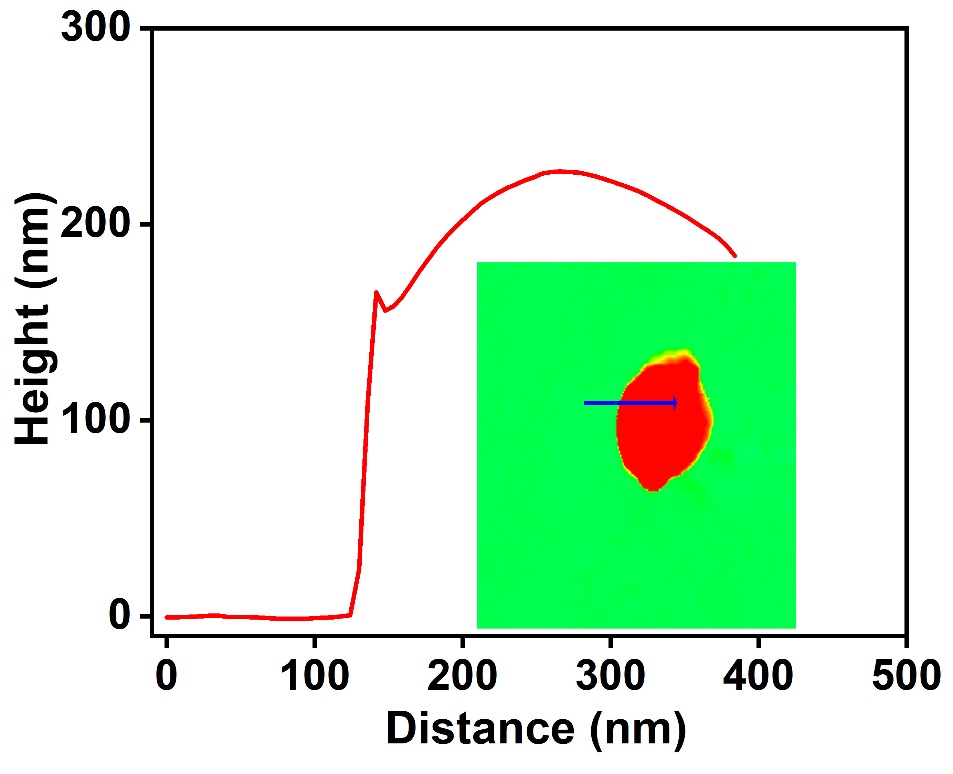


**Figure S10.** AFM image of Ru@MIP-F_7_.


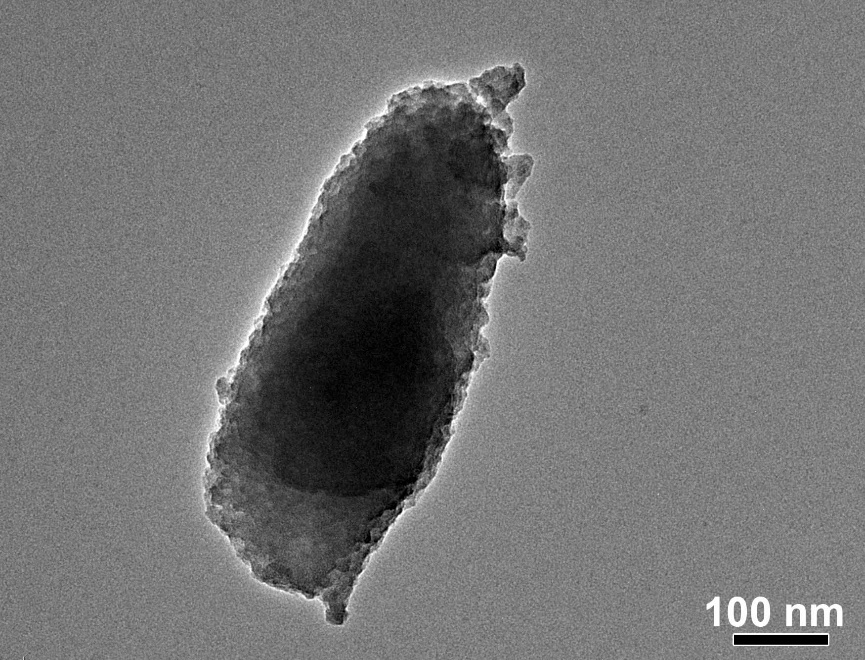


**Figure S11.** TEM image corresponding to EDX elemental mapping of Ru@MIP-F_7_.


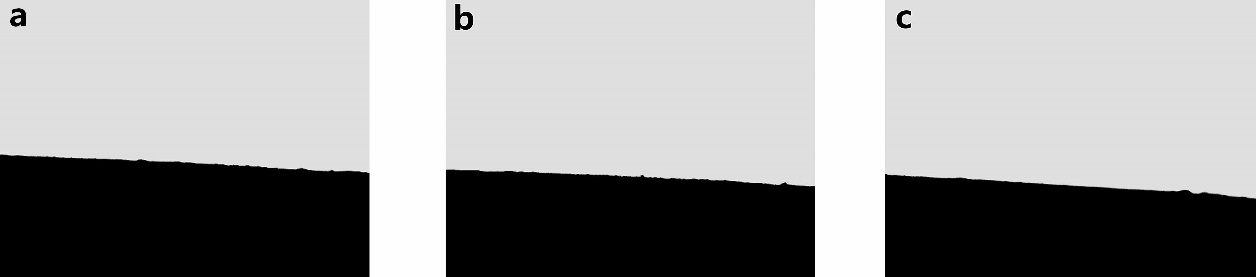


**Figure S12.** Static water contact angles of MIP-206, Ru precursor@MIP-206 and Ru@MIP-206.


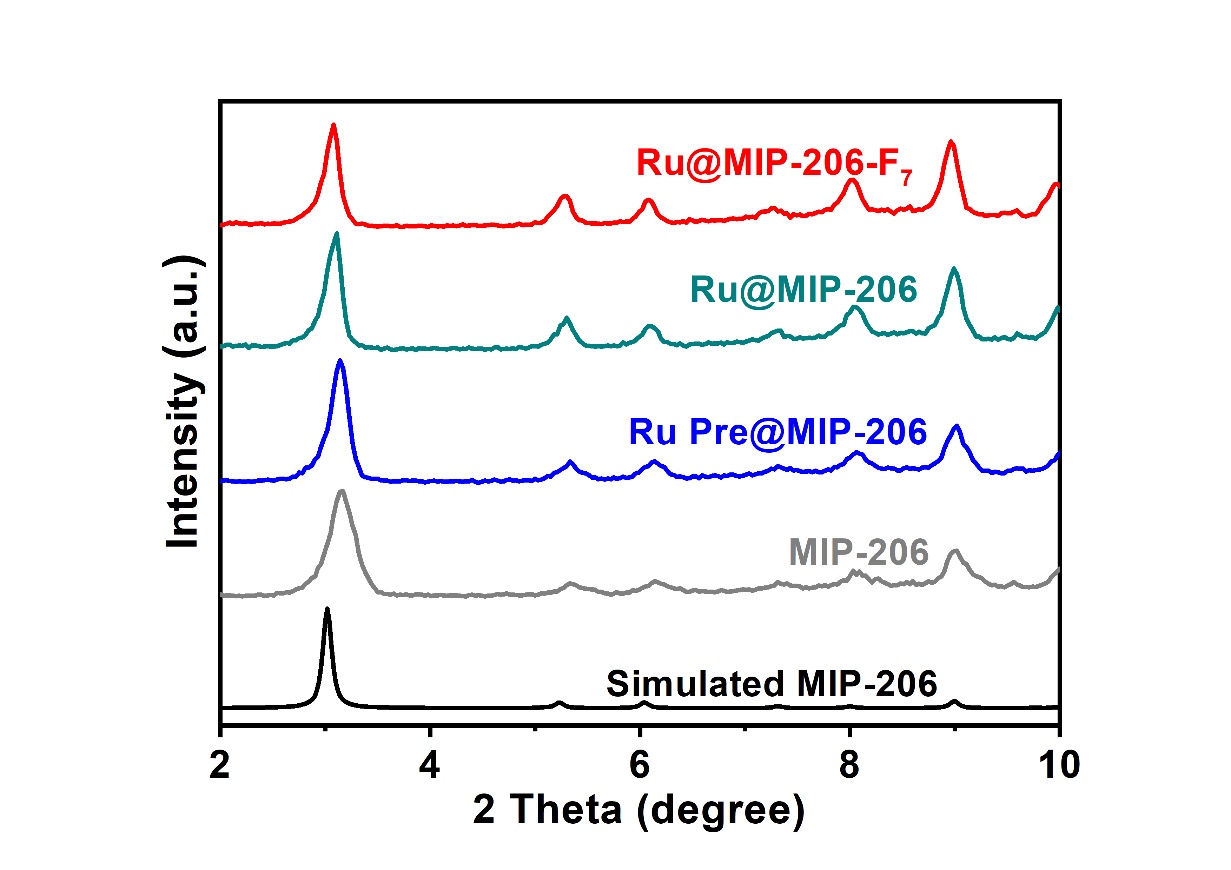


**Figure S13.** Powder XRD patterns for the as-synthesized MIP-206, Ru precusor@MIP-206, Ru@MIP-206 and Ru@MIP-F_7_.


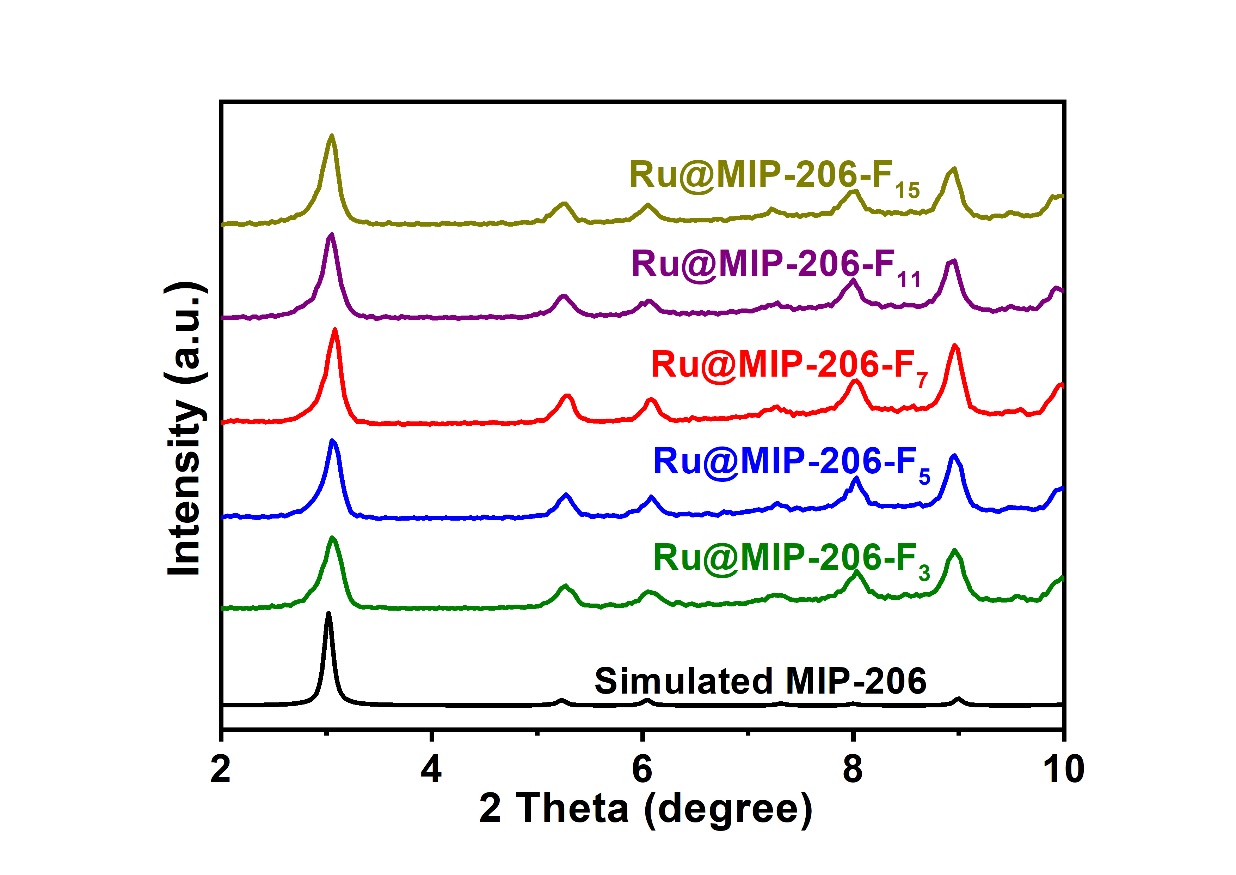


**Figure S14.** Powder XRD patterns of Ru@MIP-F_3_, Ru@MIP-F_5_, Ru@MIP-F_7_, Ru@MIP-F_11_ and Ru@MIP-F_15_.


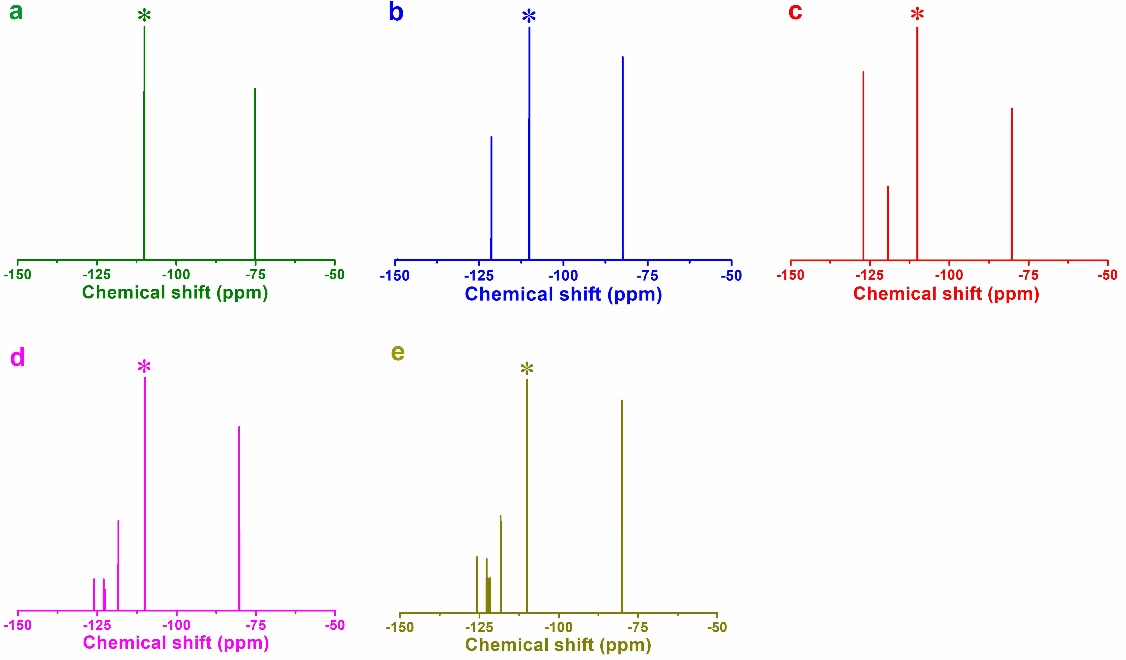


**Figure S15.** ^19^F NMR spectra of Ru@MIP-F*_x_*. ^19^F NMR spectra of Ru@MIP-F*_x_* (*x*= 3, 5, 7, 11, 15). The sample (2 mg) was digested in 10% H_2_SO_4_/DMSO-d_6_ before ^19^F NMR measurement by adding 4-fluorophenylacetylene (3 μL for Ru@MIP-F_3_ and 1 μL for Ru@MIP-F*_x_*, *x* = 5, 7, 11, 15) as an internal standard (marked with the asterisk symbol). The apparent ^19^F signals suggest that the perfluorinated alkyls are successfully grafted onto pore walls of MIP-206. The modified ratio of amino group is estimated based on the F content: Ru@MIP-F_3_ (6.1±0.9 mol%), Ru@MIP-F_5_ (5.5±1.1 mol%), Ru@MIP-F_7_ (4.8±0.8 mol%), Ru@MIP-F_11_ (5.2±0.7 mol%) and Ru@MIP-F_15_ (4.6±0.9 mol%).


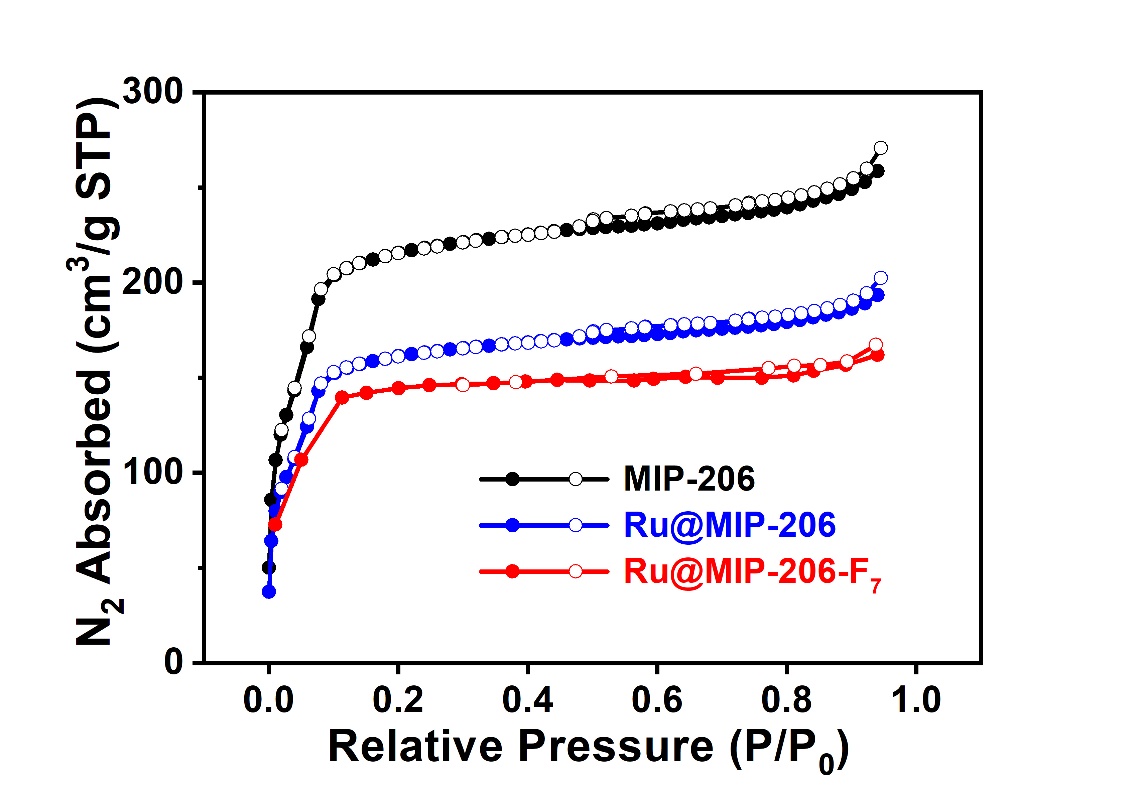


**Figure S16.** Nitrogen sorption isotherms for MIP-206, Ru@MIP-206 and Ru@MIP-F_7_ at 77 K.


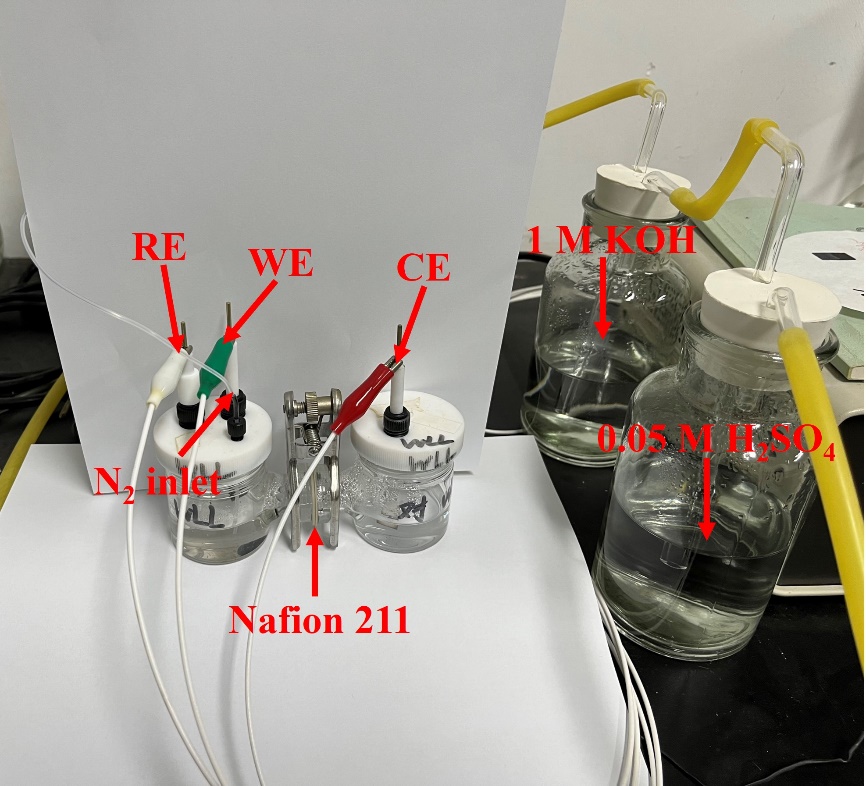


**Figure S17.** The H-cell with two compartments separated by the Nafion 211 membrane for N_2_ electroreduction.


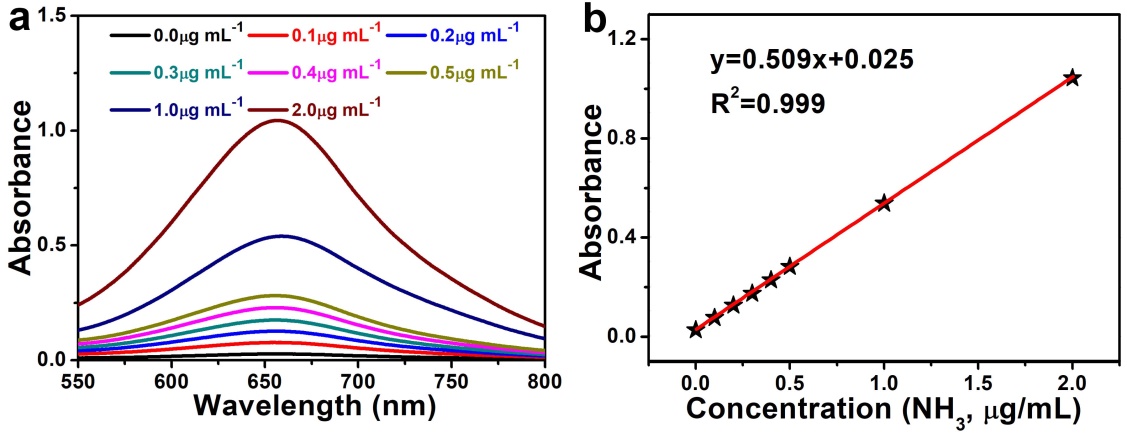


**Figure S18.** (a) UV-vis absorption spectra of indophenol assays with NH_4_^+^ ions after incubation for 2 h at room temperature. (b) Calibration curve used for estimation of NH_3_ concentration.


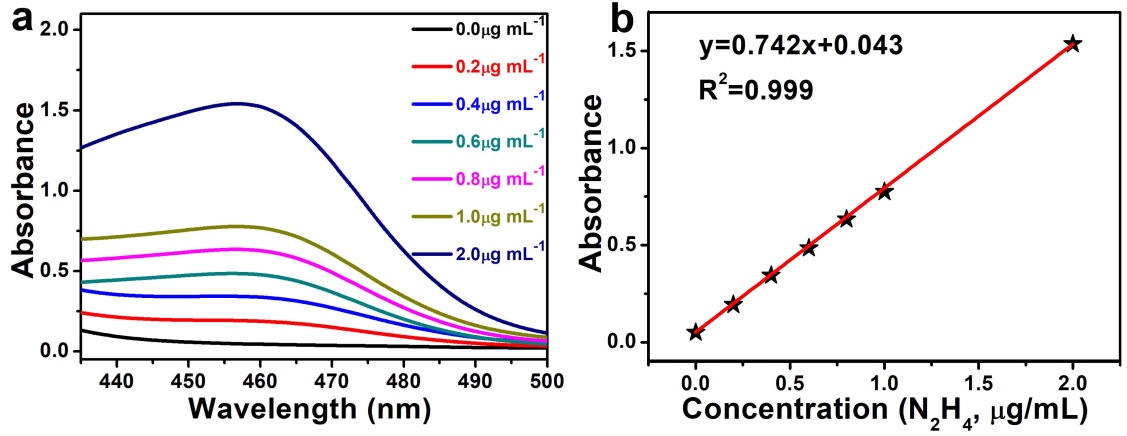


**Figure S19.** (a) UV-vis absorption spectra of various N_2_H_4_ concentrations after incubation for 15 min at room temperature. (b) Calibration curve used for calculation of N_2_H_4_ concentrations.


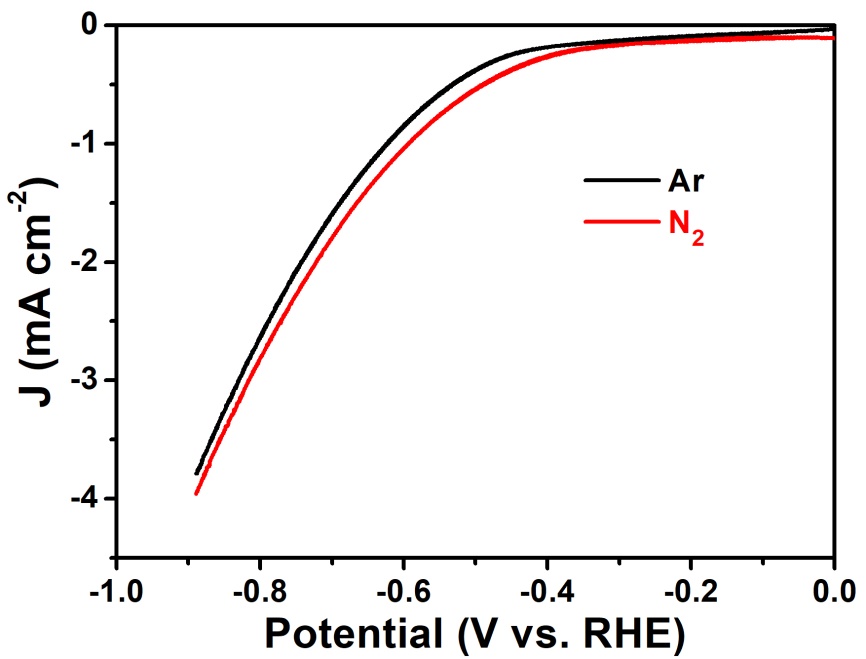


**Figure S20.** LSV curves of Ru@MIP-F_7_ in N_2_ and Ar-saturated electrolyte.


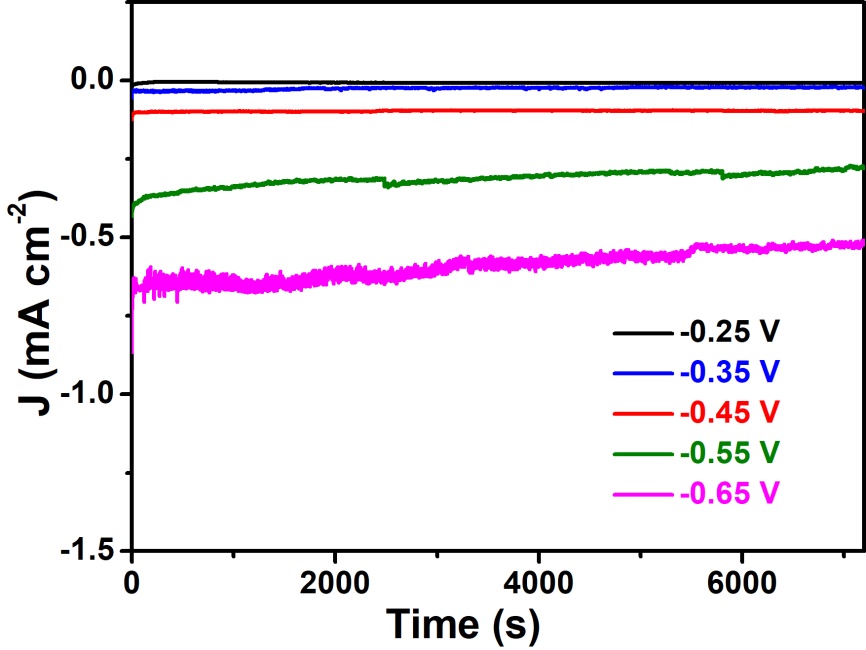


**Figure S21.** Time-dependent current density curves of Ru@MIP-F_7_ under various potentials in N_2_-saturated 0.1 M Na_2_SO_4_.


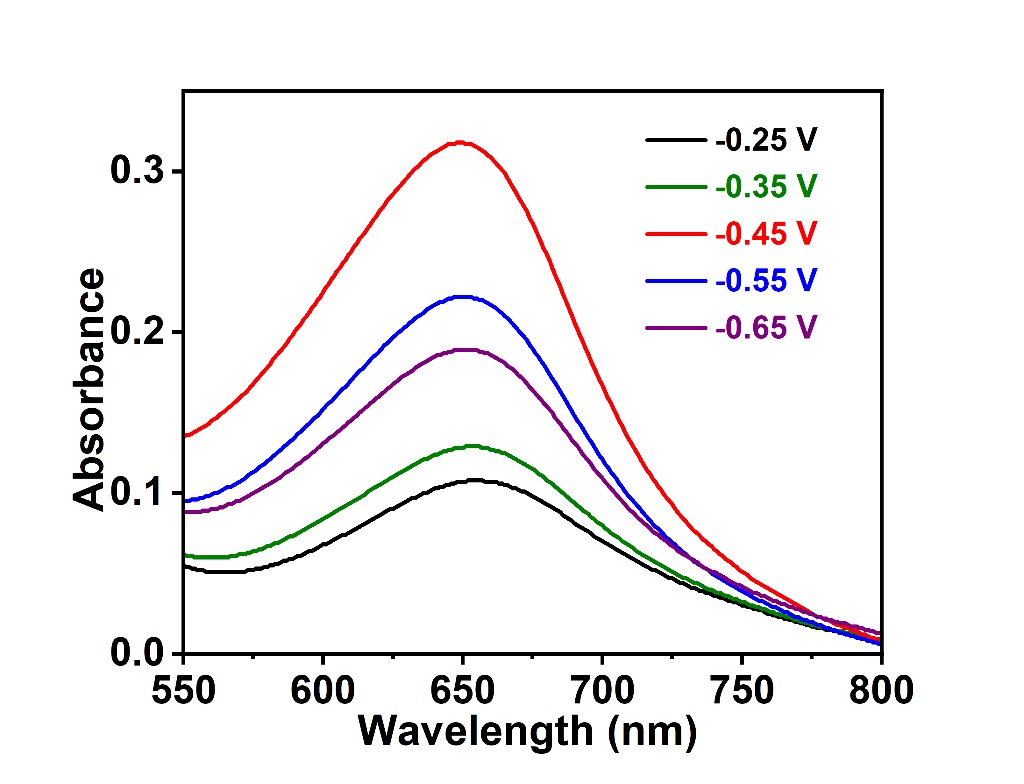


**Figure S22.** UV-vis spectra of the electrolyte at various potentials on Ru@MIP-206-F_7_.


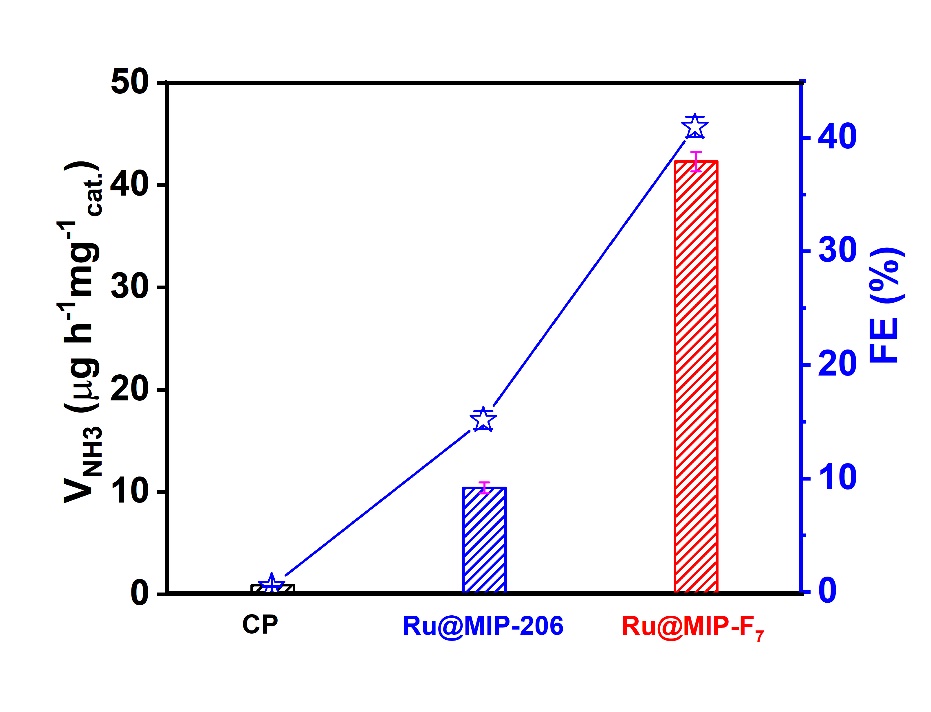


**Figure S23.** NH_3_ production rate and FE for CP, pure Ru@MIP-206, and Ru@MIP-F_7_ after 2 h of electrolysis at −0.45 V *vs.* RHE.


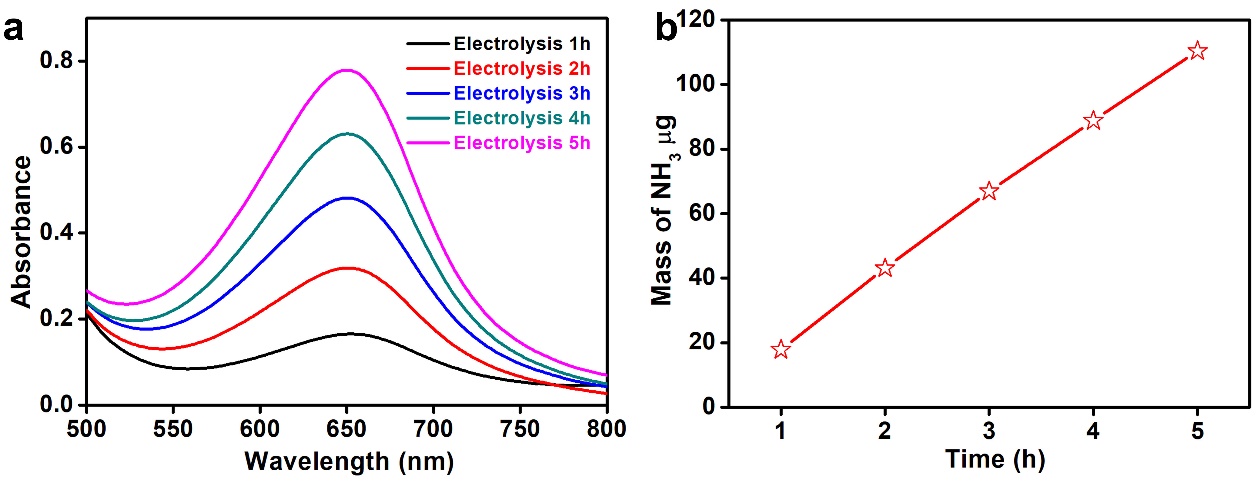


**Figure S24.** (a) UV-vis spectra of the electrolyte at various electrolysis time over Ru@MIP-F_7_. (b) The NH_3_ production of Ru@MIP-F_7_ along time.


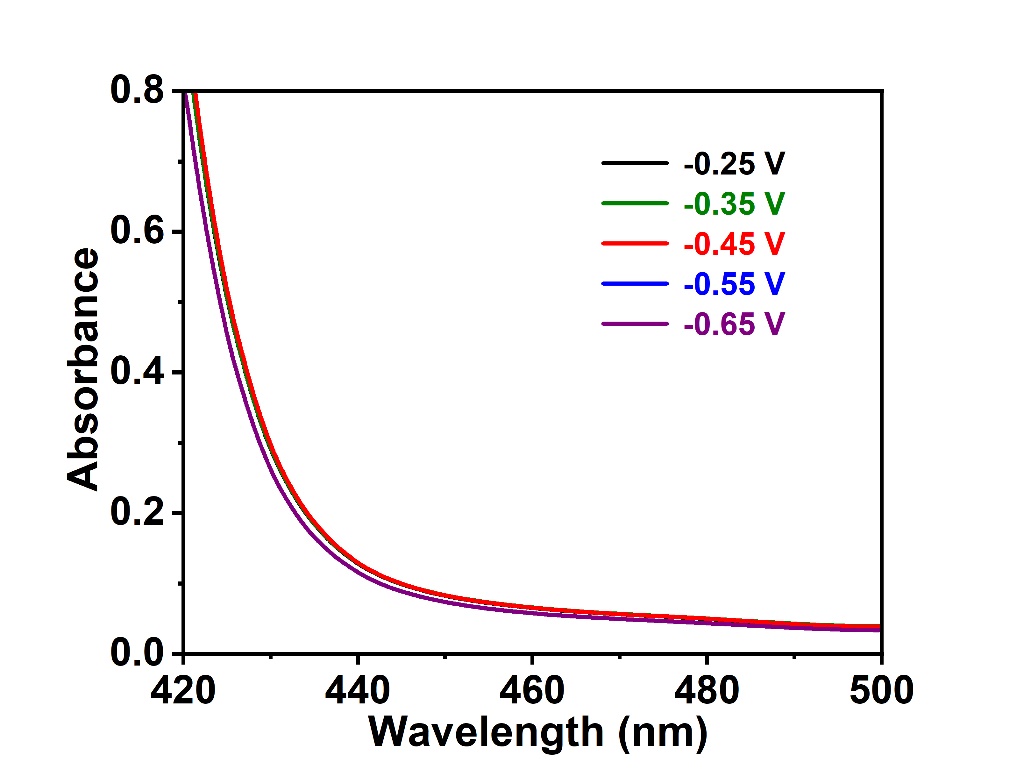


**Figure S25.** UV-vis absorption spectra of electrolytes at each given potential stained with p-C_9_H_11_NO indicator after 2h NRR electrolysis.


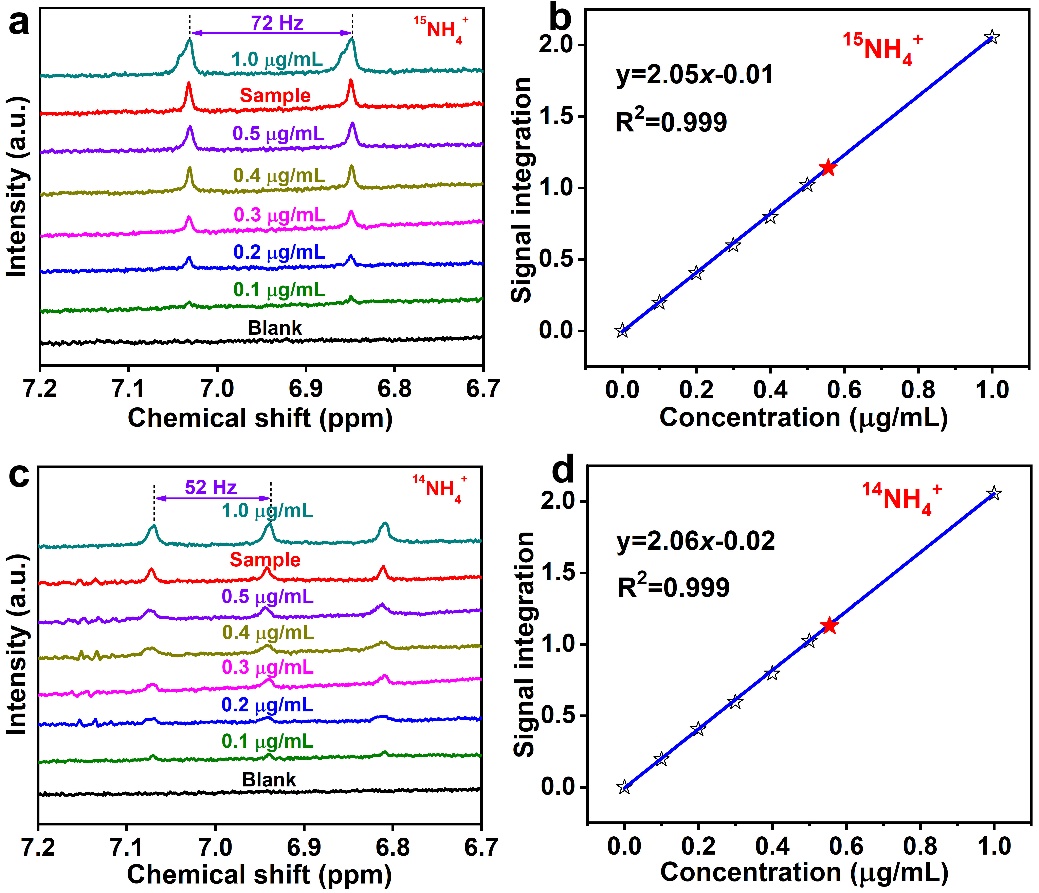


**Figure S26.** (a, b) ^1^H NMR spectra (a) and corresponding ^1^H NMR calibration curve (b) of ^15^NH_4_^+^. (c, d) ^1^H NMR spectra (c) and corresponding ^1^H NMR calibration curve (d) of ^14^NH_4_^+^. The pink asterisk indicates the NRR sample of Ru@MIP-F_7_ at −0.45 V in 0.1 M Na_2_SO_4_.


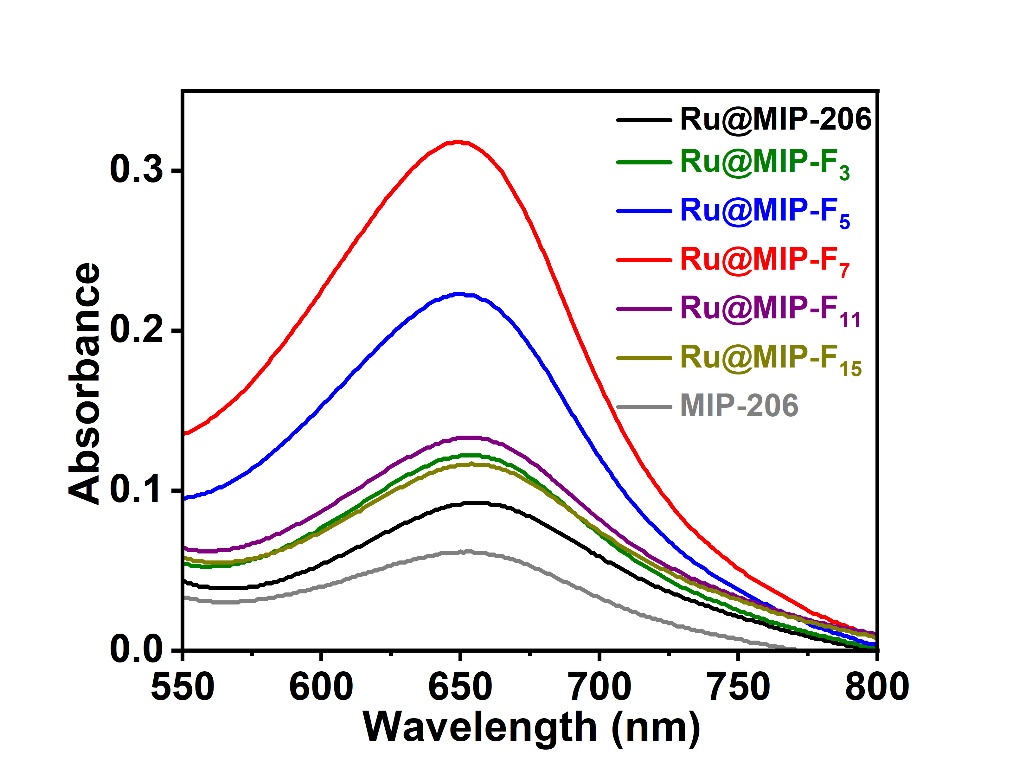


**Figure S27.** UV-vis absorption spectra of the electrolyte after electrocatalysis at -0.25 V vs. RHE for different control catalysts, including pure MIP-206, Ru@MIP-206, and Ru@MIP-F*_x_* (*x* = 3, 5, 7, 11, 15) catalysts.


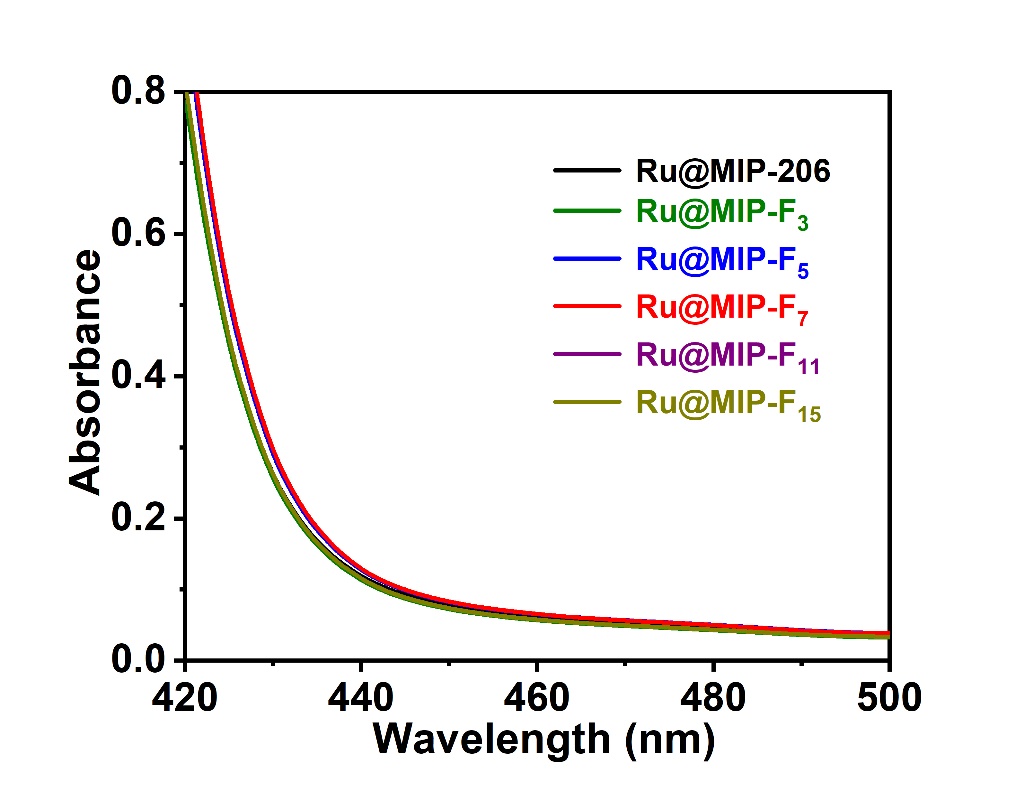


**Figure S28.** UV-vis absorption spectra of electrolytes at each given potential stained with p-C_9_H_11_NO indicator after 2h NRR electrolysis.


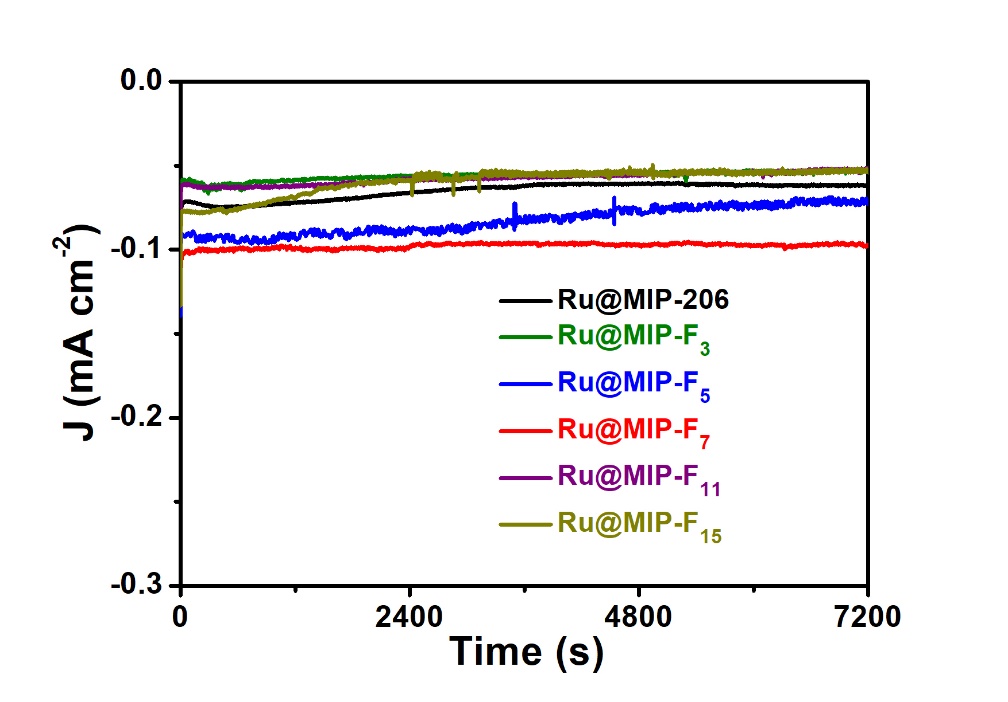


**Figure S29.** Time-dependent current density curves of Ru@MIP-206 and Ru@MIP-F*_x_* (*x* = 3, 5, 7, 11, 15) at −0.45 V *vs.* RHE in N_2_-saturated 0.1 M Na_2_SO_4_.


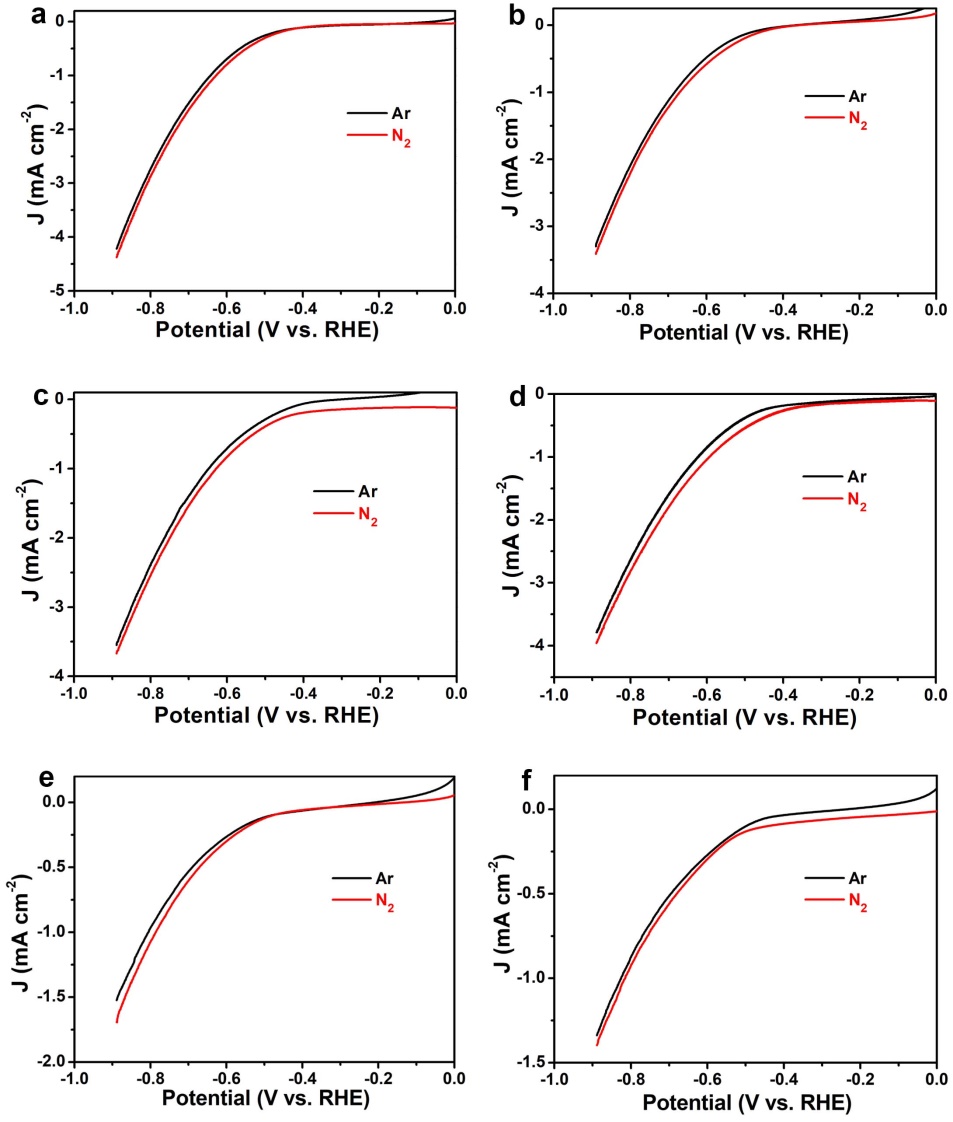


**Figure S30.** LSV curves of (a) Ru@MIP-206, (b) Ru@MIP-F_3_, (c) Ru@MIP-F_5_, (d) Ru@MIP-F_7_, (e) Ru@MIP-F_11_, (f) Ru@MIP-F_15_ in Ar- and N_2_-saturated 0.1 M Na_2_SO_4_.


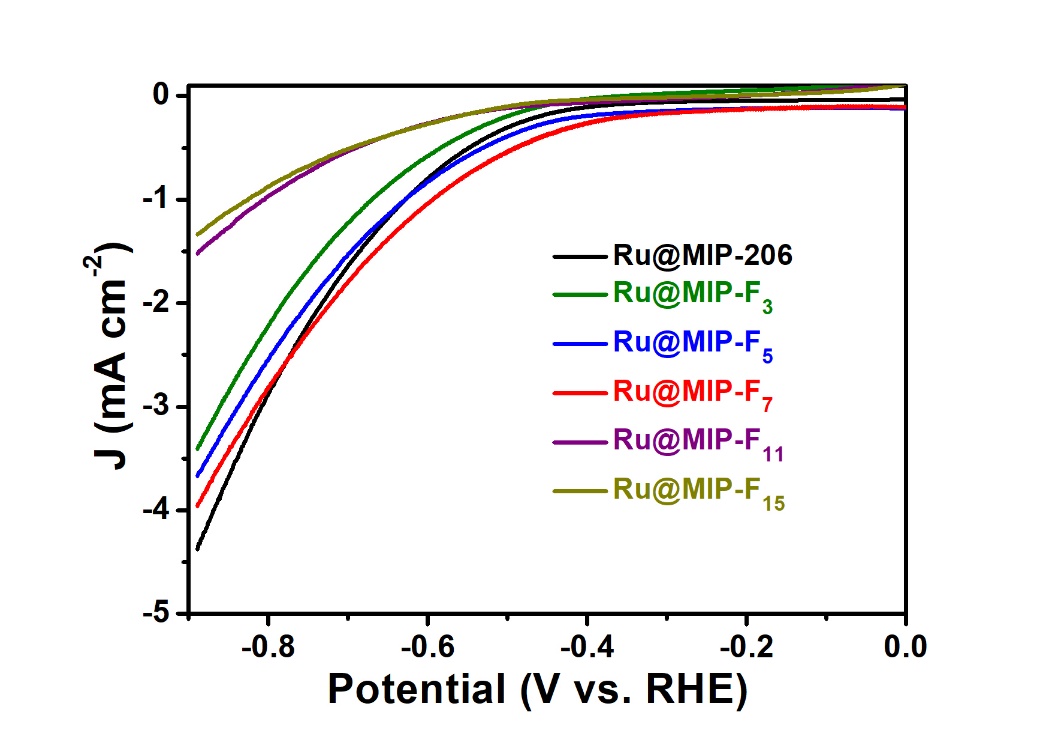


**Figure S31.** Comparison of LSV curves of Ru@MIP-206 and Ru@MIP-F*_x_* (*x* = 3, 5, 7, 11, 15) in N_2_-saturated 0.1 M Na_2_SO_4_ solution.


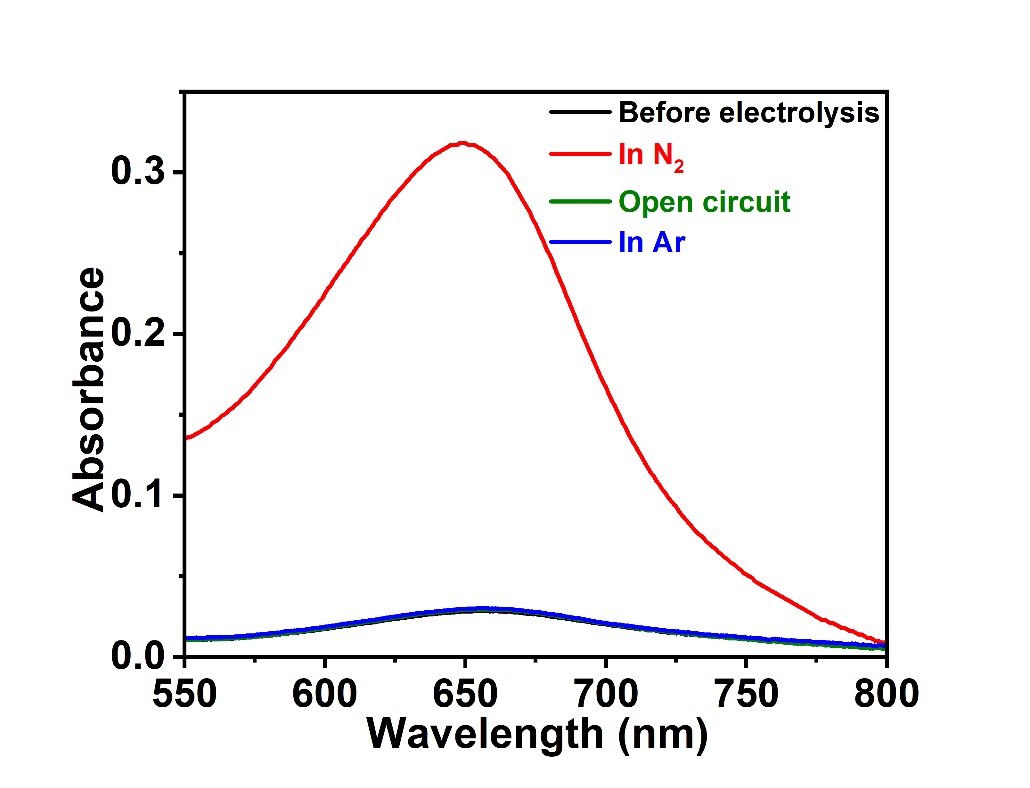


**Figure S32.** UV-vis absorption spectra of the electrolytes stained with indophenol indicator after 2 h electrolysis at –0.45 V under different electrochemical conditions.


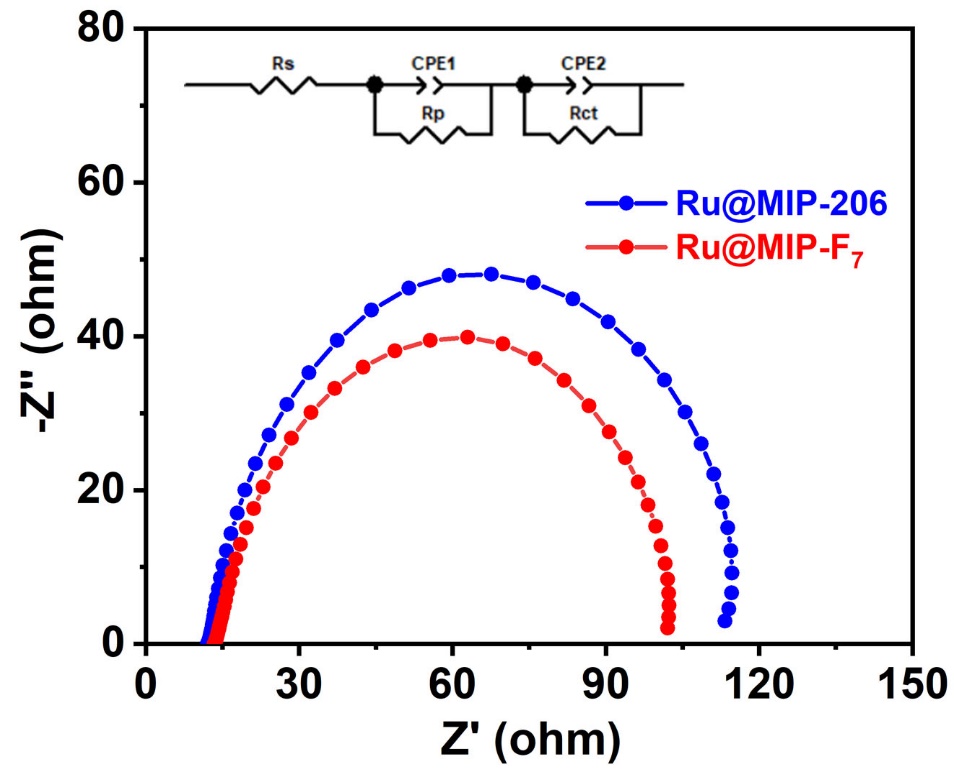


**Figure S33.** EIS Nyquist plots and equivalent circuit diagram of Ru@MIP-206 and Ru@MIP-F_7_.


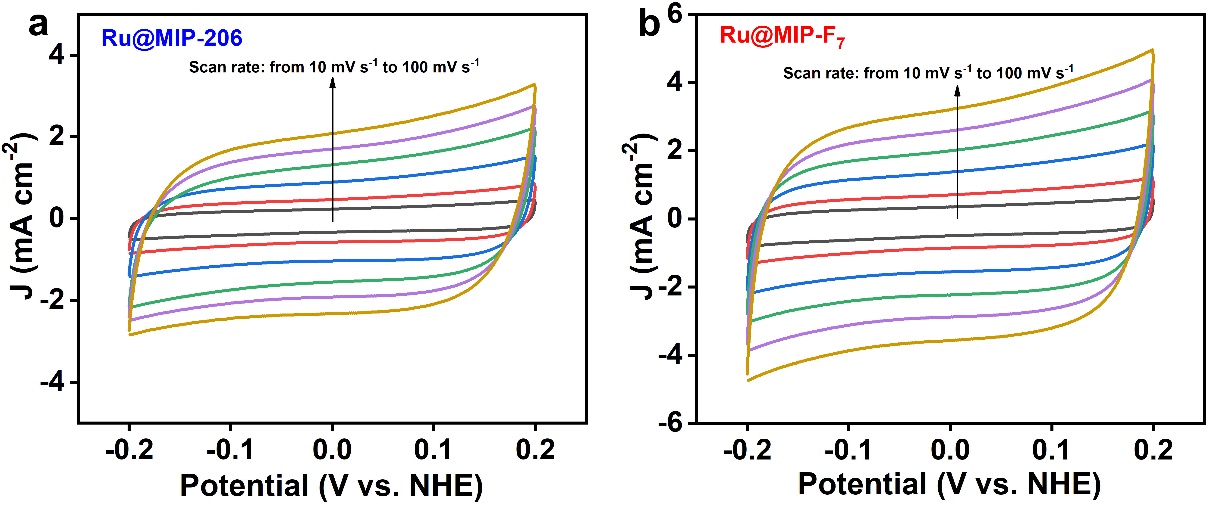


**Figure S34.** Cyclic voltammograms curves were performed at various scan rates on (a) Ru@MIP-206 and (b) Ru@MIP-F_7_.


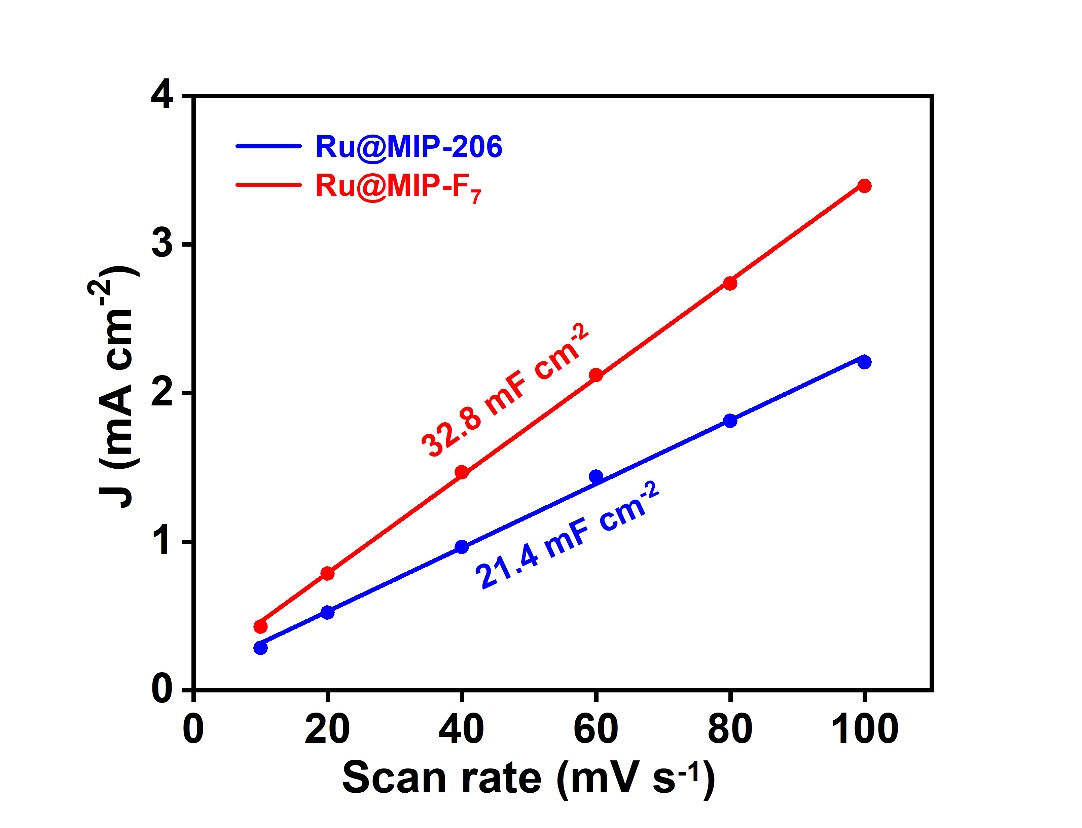


**Figure S35.** Linear fittings of the capacitive current densities at different scan rates of Ru@MIP-206 and Ru@MIP-F_7_.


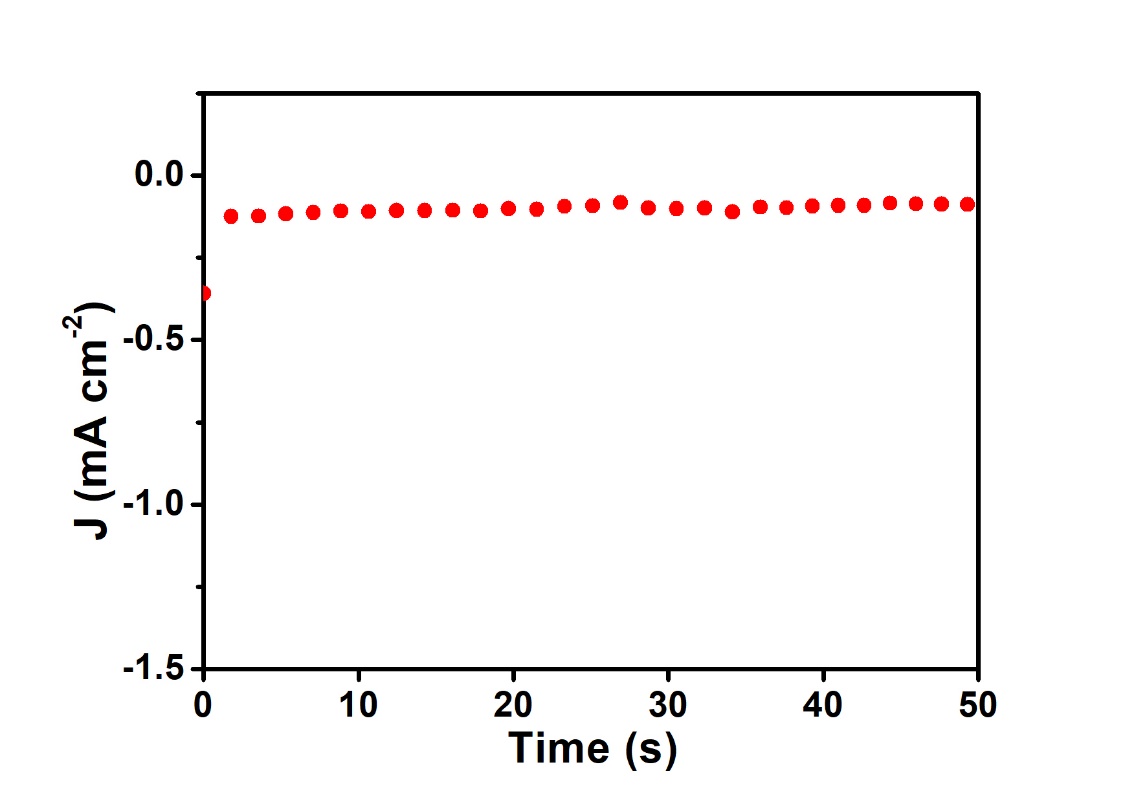


**Figure S36.** Time-dependent current density during electrolysis at -0.45 V *vs.* RHE for 50 h.


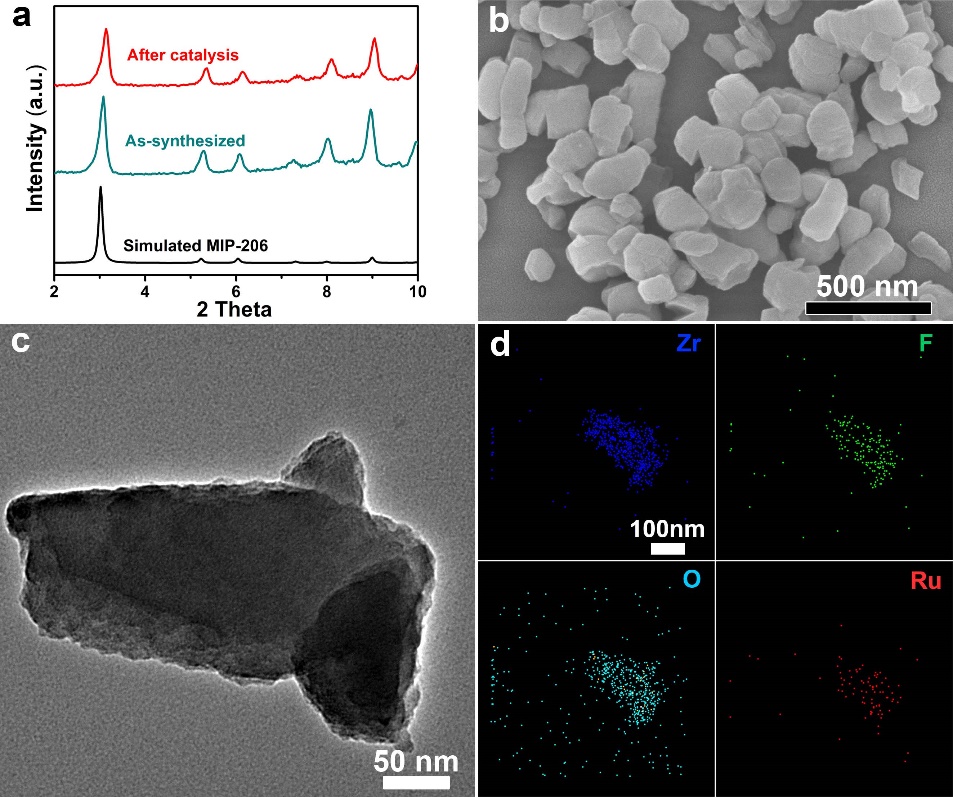


**Figure S37.** (a) XRD patterns of Ru@MIP-F_7_ before and after stability test in 0.1M Na_2_SO_4_ solution. (b) SEM, (c) TEM and (d) elemental mapping images of Ru@MIP-F_7_ after stability test.


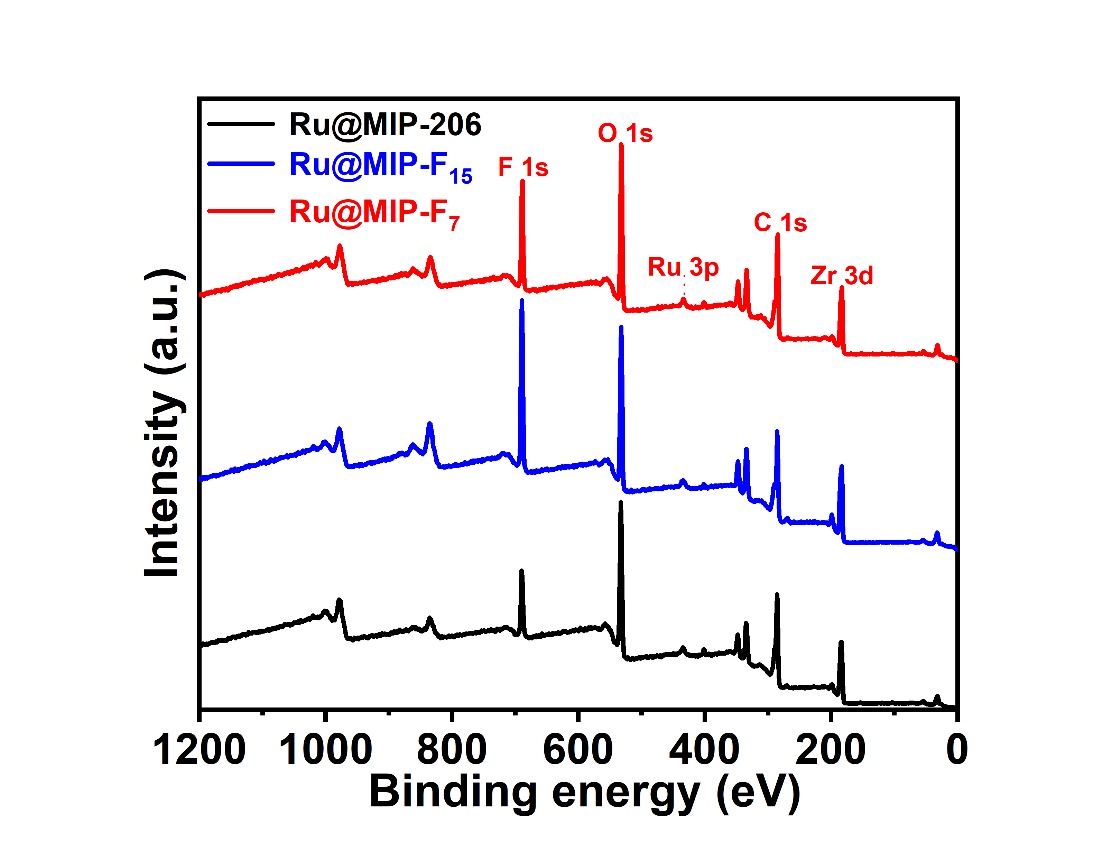


**Figure S38.** XPS survey spectrum of Ru@MIP-206, Ru@MIP-F_15_ and Ru@MIP-F_7_.


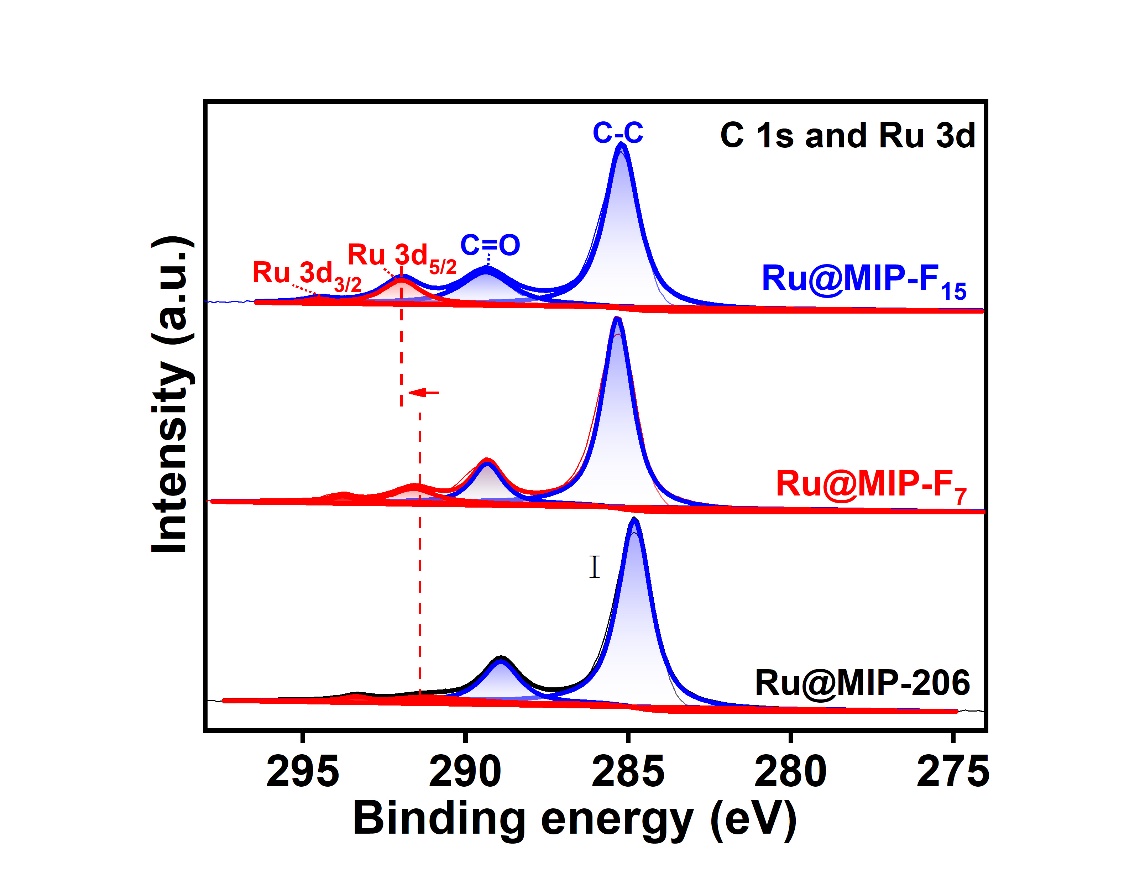


**Figure S39.** The high-resolution XPS of C 1s and Ru 3d of Ru@MIP-206, Ru@MIP-F_15_ and Ru@MIP-F_7_.


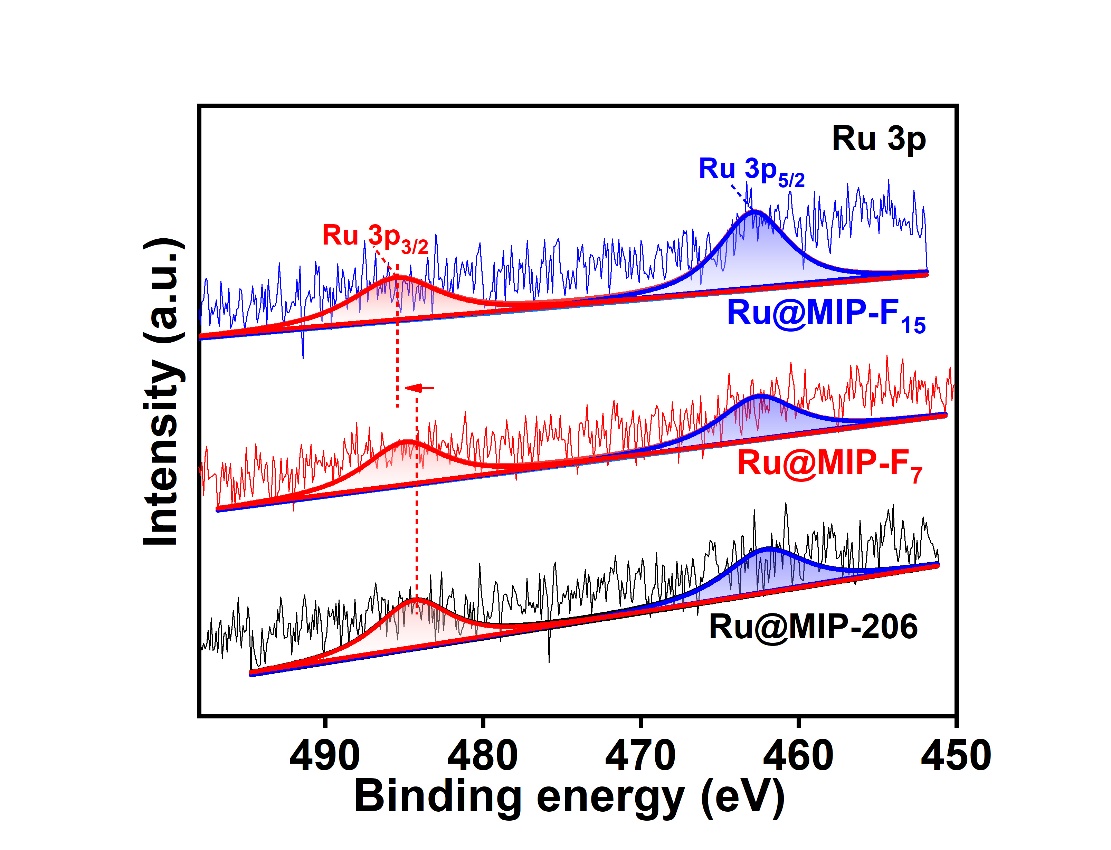


**Figure S40.** The high-resolution XPS of Ru 3p of Ru@MIP-206, Ru@MIP-F_15_ and Ru@MIP-F_7_.


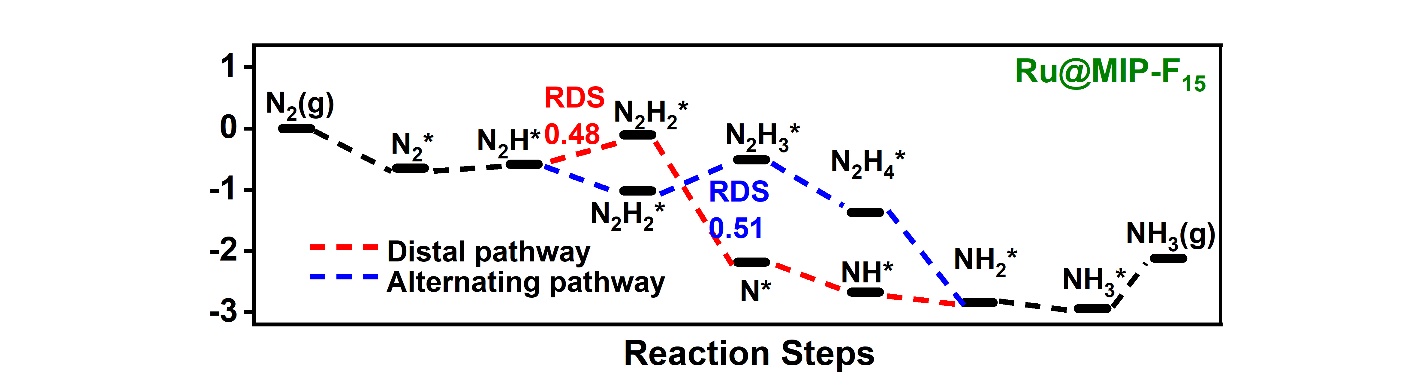


**Figure S41.** The energy variations of NRR process along reaction path of Ru@MIP-F_15_.


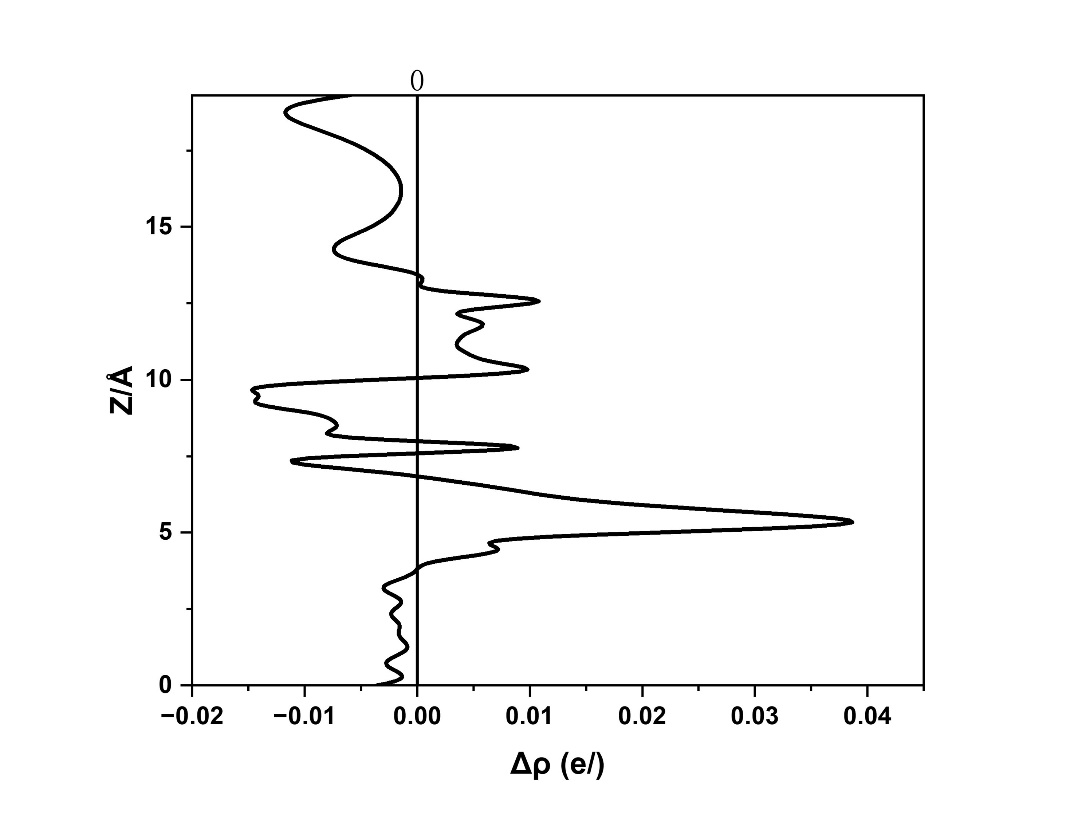


**Figure S42.** The PDOS of Ru@MIP-F_7_ catalyst.


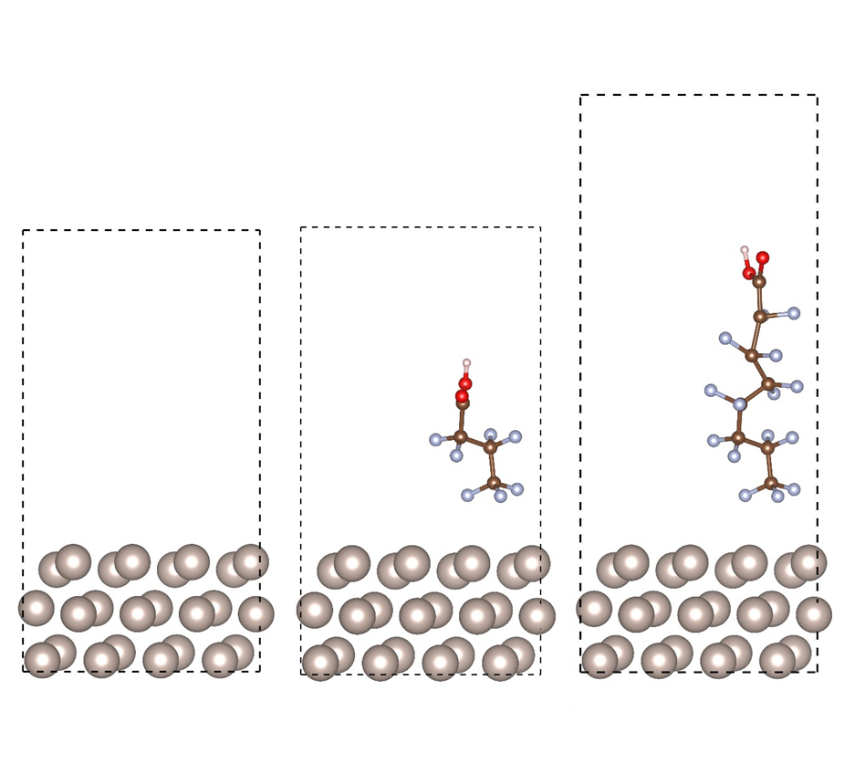


**Figure S43.** The optimized models of catalysts, where ruthenium, grey, red, white, and blue balls represent Ru, C, O, H, and F atom, respectively.

**Table S1.** The Ru content in Ru@MIP-206 and Ru@MIP-F*_x_* (*x* = 3, 5, 7, 11, 15) samples.

| **Entry** | **Catalyst** | **Amount of Ru (wt %)** |
| --- | --- | --- |
| 1 | Ru@MIP-206 | 2.24 |
| 2 | Ru@MIP-F_3_ | 1.75 |
| 3 | Ru@MIP-F_5_ | 1.93 |
| 4 | Ru@MIP-F_7_ | 1.89 |
| 5 | Ru@MIP-F_11_ | 1.86 |
| 6 | Ru@MIP-F_15_ | 1.79 |

The data are based on inductively coupled plasma atomic emission spectrometer (ICP-AES).

**Table S2.** Comparison of the electrocatalytic NRR performance of Ru@MIP-F_7_ with other previously reported NRR electrocatalysts.

| **Catalyst** | **Electrolyte** | **NH_3_ yield rate** | **FE** | **References** |
| --- | --- | --- | --- | --- |
| **Ru@MIP-F_7_** | **0.1 M Na_2_SO_4_** | **42.29 μg h^-1^ mg_cat._^-1^** | **40.94%** | **This work** |
| Ru/rGO | 0.05M H_2_SO_4_ | 50 μg h^-1^ mg_cat._^-1^ | 11% | *Angew. Chem. Int. Ed.* **2020,** *59,* 21465-21469 |
| Ru SAs/N-C | 0.05M H_2_SO_4_ | 120.9 μg h^-1^ mg_cat._^-1^ | 29.6% | *Adv. Mater.* **2018,** *30,* 1803498 |
| Ru_2_P-rGO | 0.1 M HCl | 32.8 μg h^-1^ mg_cat._^-1^ | 13.04% | *J. Mater. Chem. A,* **2020,** *8,* 77-81 |
| Ru@ZrO_2_/NC | 0.1 M HCl | 3.665 mg h^-1^ mg_cat._^-1^ | 21% | *Chem* **2019,** *5,* 204-214 |
| Ru-Mo_2_CT*x* | 0.5 M K_2_SO_4_ | 40.57 µg h^-1^ cm^-2^ | 25.77% | *Adv. Energy Mater.* **2020,** *10,* 2001364 |
| Ru_0.15_Cu_0.85_ | 0.1 M KOH | 26.25 μg h^-1^ mg_cat._^-1^ | 4.39% | *ACS Appl. Mater. Interfaces* **2023,** *15,* 11703-11712 |
| Ru-Cu NPs | 0.1 M HCl | 73 µmol h^−1^ cm^−2^ | 31% | *Adv. Mater.* **2022,** *34,* 2205270 |
| LaFeO-Ru | 0.5M H_2_SO_4_ | 137.5 μg h^-1^ mg_cat._^-1^ | 56.9% | *Small* **2023,** *19,* 2208102 |
| Ru–Mn_3_O_4_ | 0.1 M Na_2_SO_4_ | 35.34 μg h^-1^ mg_cat._^-1^ | 28.87% | *Adv. Mater.* **2022,** *34,* 2108180 |
| Ru/Ti_3_C_2_O | 0.1 M HCl | 27.56 μg h^-1^ mg_cat._^-1^ | 23.3% | *ChemSusChem* **2022,** *15,* e202102352 |

**References**

[S1] S. Wang, L. Chen, M. Wahiduzzaman, A. Tissot, L. Zhou, I. A. Ibarra, A. Gutiérrez-Alejandre, J. S. Lee, J.-S. Chang, Z. Liu, J. Marrot, W. Shepard, G. Maurin, Q. Xu, C. Serre, *Matter* **2021**, *4*, 182.

[S2] L. Li, Z. Li, W. Yang, Y. Huang, G. Huang, Q. Guan, Y. Dong, J. Lu, S.-H. Yu, H.-L. Jiang, *Chem* **2021**, *7*, 686.

[S3] D. Chen, W. Yang, L. Jiao, L. Li, S. H. Yu, H. L. Jiang, *Adv. Mater.* **2020**, *32*, 2000041.

[S4] X. Chen, Y. Guo, X. Du, Y. Zeng, J. Chu, C. Gong, J. Huang, C. Fan, X. Wang, J. Xiong, *Adv. Energy Mater.* **2019**, *10*, 1903172.

[S5] S. Chung, H. Ju, M. Choi, D. Yoon, J. Lee, *Angew. Chem. Int. Ed.* **2022**, *61*, e202212676.

[S6] Y. Fang, Y. Xue, L. Hui, H. Yu, Y. Li, *Angew. Chem. Int. Ed.* **2021**, *60*, 3170.

[S7] S. Grimme, J. Antony, S. Ehrlich, H. Krieg, *J. Chem. Phys.* **2010**, *132*, 154104.

[S8] J. P. Perdew, K. Burke, M. Ernzerhof, *Phys. Rev. Lett* **1988,** *77,* 3865.

[S9] G. Kresse, J. Furthmüller, *Comp. Mater. Science* **1996,** *6,* 15.

[S10] J. K. Norskov, J. Rossmeisl, A. Logadottir, L. Lindqvist, J. R. Kitchin, T. Bligaard, H. Jónsson, *J. Phys. Chem. B* **2004,** *108,* 17886.

[S11] V. Wang, N. Xu, J.-C. Liu, G. Tang, W.-T. Geng, *Comput. Phys. Commun.* **2021**, *267*.

[S12] Z. Geng, Y. Liu, X. Kong, P. Li, K. Li, Z. Liu, J. Du, M. Shu, R. Si, J. Zeng, *Adv. Mater.* **2018**, *30*, 1803498.

[S13] Y. Luo, M. Li, Y. Dai, X. Zhang, R. Zhao, F. Jiang, C. Ling, Y. Huang, *J. Mater. Chem. A* **2021**, *9*, 15217.
